# A Promising MM–Fe–Al–Ga–B Permanent Material with Substantially Boosted Magnetic Performance

**DOI:** 10.1002/advs.202417679

**Published:** 2025-04-17

**Authors:** Wang Chen, Shaoqing Ren, Jiaying Jin, Liang Zhou, Bo Xin, Mingjing Zhao, Mengfan Bu, Xu Li, Chen Wu, Mi Yan

**Affiliations:** ^1^ State Key Laboratory of Baiyunobo Rare Earth Resource Researches and Comprehensive Utilization Baotou Research Institution of Rare Earths Baotou 014030 China; ^2^ State Key Laboratory of Silicon and Advanced Semiconductor Materials School of Materials Science and Engineering Key Laboratory of Novel Materials for Information Technology of Zhejiang Province Zhejiang University Hangzhou 310027 China

**Keywords:** Al‐doping, grain boundary diffusion processing, mischmetal, RE_6_(Fe, Al, Ga)_14_ phase, REFe_2_ phase

## Abstract

Exploiting the potential of mischmetal (MM, La–Ce–Pr–Nd mixture containing ≈80 wt% La–Ce) based MM–Fe–B permanent material as an advantageous alternative for the current Nd–Fe–B is intriguing, whereas remains an arduous quest due to the drastically deteriorated magnetic performance. Herein, a facile two‐step strategy is proposed that Al‐doping towards high maximum energy product (BH)_max_ with the subsequential grain boundary diffusion processing (GBDP) toward high coercivity *H*_cj_, i.e., a representative 0.7 wt% Al doping and Nd/Pr‐based GBDP yields the record‐high *H*_cj_ = 10.14 kOe and (BH)_max_ = 28.02 MGOe for the MM–Fe–Al–Ga–B sintered magnets. Appropriate Al‐doping plays a pivotal role in accelerating the La/Ce segregation in the LaCe‐rich RE–(Al, Ga), La‐rich RE_6_(Fe, Al, Ga)_14_, and Ce‐rich REFe_2_ intergranular phases, resulting in enhanced intrinsic and extrinsic magnetism. The following Nd/Pr‐based GBDP generates large quantities of RE_6_(Fe, Al, Ga)_14_ phase in triple junctions and grain boundaries, together with continuous Nd/Pr‐rich magnetically hardening shell, finally harvesting the substantially boosted magnetic performance. This demonstration marks a clear picture of the two‐step strategy towards fully utilizing the abundant MM resources, and overcoming the long‐standing difficulty of applicable MM–Fe–B permanent material for industrialization.

## Graphical abstract

A facile two‐step strategy, Al‐doping toward high‐(BH)_max_ followed by Nd/Pr‐based grain boundary diffusion processing toward high‐*H*_cj_, endows the microcrystalline MM–Fe–Al–Ga–B magnet with substantially boosted magnetic performance [(BH)_max_ = 28.02 MGOe, *H*_cj_ = 10.14 kOe]. These previously unattainable magnetic properties explicitly demonstrate the promising commercial prospects of rationally designed MM–Fe–Al–Ga–B permanent material.

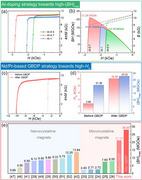

## 1 Introduction

Unique 4f electronic structure endows the rare earth (RE) elements with strong spin–orbit interaction and large magnetocrystalline anisotropy field (*H*_A_), furnishing a potent stimulus in discovering superb RE‐based permanent magnets. One important embodiment is the strongest 2:14:1 type RE–Fe–B permanent material with the largest annual RE consumption among wide‐ranged applications.^[^ ^1^, ^2^, ^3^ ^]^ In recent years, the continually evolving technologies such as electric vehicles and high‐tech electronics pertinent to energy efficiency trigger escalating demand for RE–Fe–B permanent material, necessitating more efficient utilization of valuable RE resources.^[^ ^4^, ^5^ ^]^ In this context, exploiting the potential of abundant and inexpensive RE elements including La/Ce to prepare the La/Ce‐rich RE–Fe–B magnet as an advantageous alternative for the conventional Nd–Fe–B appears as a focal research point.^[^ ^6^, ^7^ ^]^ Currently, the Nd–Ce–Fe–B or the Nd–La–Ce–Fe–B sintered magnet containing 30–40 wt% La/Ce has emerged as a promising candidate in view of the preferable magnetic properties, i.e., maximum energy product (*BH*)_max_ of 40.57 MGOe for the 40 wt% Ce substituted Nd–Ce–Fe–B magnet,^[^ ^8^ ^]^ coercivity *H*_cj_ of 13.75 kOe for the 35 wt% LaCe substituted Nd–La–Ce–Fe–B magnet.^[^ ^9^ ^]^ Nevertheless, the magnetic performance at higher La/Ce substitution levels is still ungratified due to the coercivity limit, which is usually below 10 kOe for the 50 wt% Ce or LaCe substituted bulk magnets.^[^ ^10^, ^11^, ^12^, ^13^ ^]^

**Figure 1:**
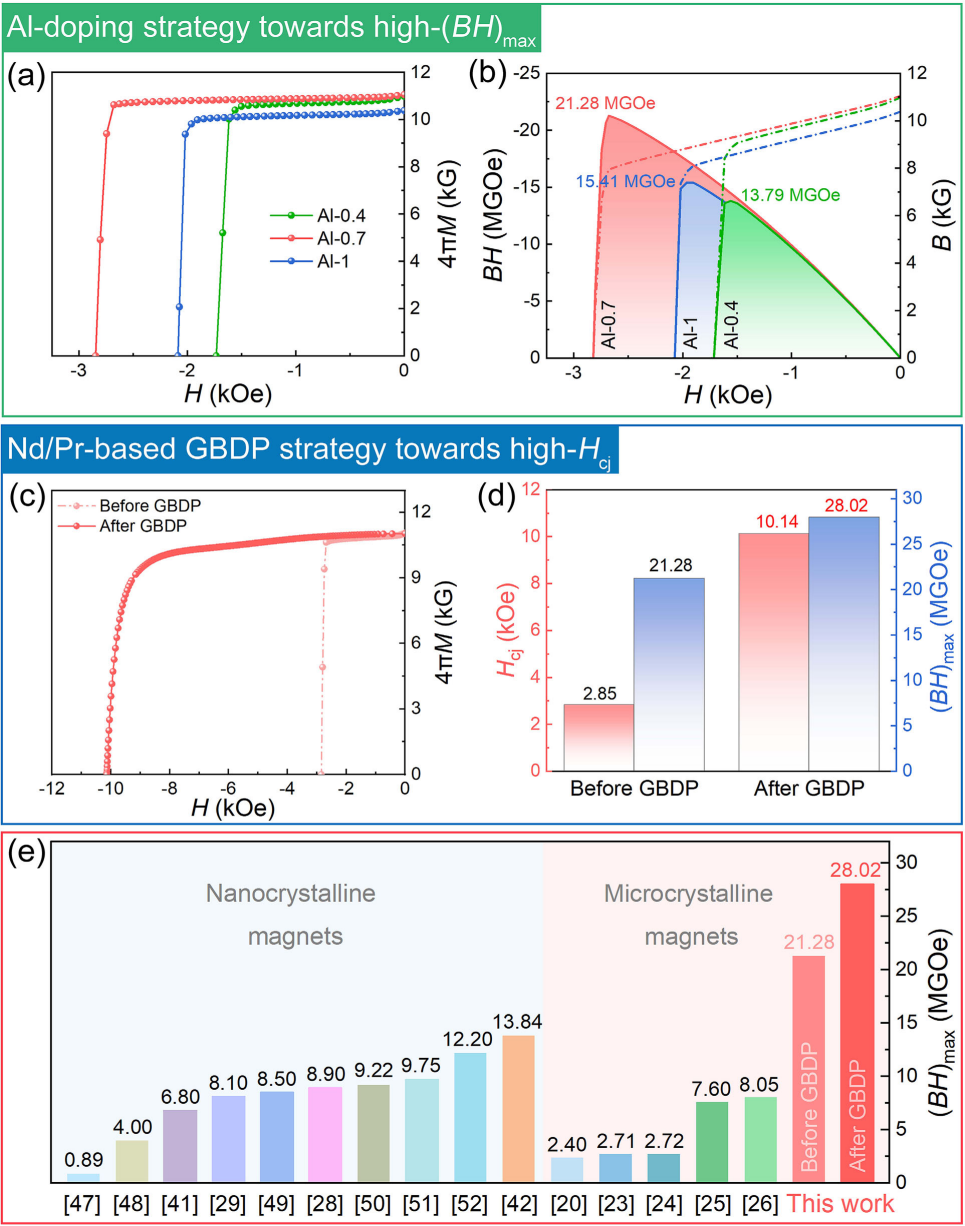
Substantially boosted magnetic performance of the MM–Fe–Al–Ga–B magnets via two‐step strategy. Al‐doping strategy towards high‐(*BH*)_max_: a) *J* *–* *H*, b) *B*–*H* demagnetization curves of the Al‐0.4, Al‐0.7 and Al‐1 magnets. Subsequential Nd/Pr‐based GBDP strategy towards high‐*H*_cj_: c) demagnetization curves, d) derived *H*_cj_ and (*BH*)_max_ values of the MM–Fe–Al–Ga–B magnets before and after GBDP. e) Benchmark (*BH*)_max_ of the present Al‐0.7 magnet before and after GBDP (marked by red columns) compared with a wide range of other nanocrystalline^[^ ^28^, ^29^, ^41^, ^42^, ^47^, ^48^, ^49^, ^50^, ^51^, ^52^ ^]^ and microcrystalline^[^ ^20^, ^23^, ^24^, ^25^, ^26^ ^]^ MM–Fe–B magnets, showcasing the exceptional (*BH*)_max_ values in present work.

The utilization of RE mixture mischmetal (denoted as MM, a combination of La–Ce–Pr–Nd metal containing ≈80 wt% La–Ce) with less separation cost and environmental pollution creates a definite advantage over pure La or Ce. The attempts of MM‐based MM–Fe–B permanent material can be traced back to the late 1980s.^[^ ^14^, ^15^ ^]^ However, the pathways for realizing promising magnetic properties in MM–Fe–B sintered magnets catering to the practical application remain an arduous quest. Due to the inferior intrinsic magnetism of La_2_Fe_14_B (*H*_A_ = 20 kOe, *J*_s_ = 13.8 kG) and Ce_2_Fe_14_B (*H*_A_ = 26 kOe, *J*_s_ = 11.7 kG) compounds than that of Nd_2_Fe_14_B (*H*_A_ = 73 kOe, *J*_s_ = 16.0 kG),^[^ ^16^ ^]^ the high La–Ce concentration of ≈80 wt% in MM degrades the intrinsic magnetic properties of MM–Fe–B sintered magnets.^[^ ^17^, ^18^ ^]^ From the extrinsic perspective, due to the strong oxidation tendency of vulnerable La/Ce, the instability of La_2_Fe_14_B phase, and the diffusion of multiple RE elements at high sintering temperatures, the easier formation of paramagnetic or soft‐magnetic phases (such as REO_x_,^[^ ^19^ ^]^ REFe_2_,^[^ ^20^ ^]^ Fe‐rich phase,^[^ ^21^ ^]^ and unclear grain boundaries^[^ ^22^ ^]^) and resultantly deteriorated microstructure severely lower the magnetic properties of MM–Fe–B sintered magnets. As a consequence, available publications reveal the *H*_cj_ of merely 0–1.5 kOe for the microcrystalline MM–Fe–B sintered magnets (Figure S1, Supporting Information).^[^ ^20^, ^23^, ^24^, ^25^, ^26^ ^]^ Comparably, a larger coercivity can be achieved in the nanocrystalline MM–Fe–B melt‐spun magnets, since a non‐equilibrium process of fast cooling can suppress the formation of unfavorable secondary phases and yield refined grain size.^[^ ^27^ ^]^ For example, the coercivity is 5.6 kOe in the stoichiometric MM_12_Fe_82_B_6_ melt‐spun alloy, increases to 9.4 kOe in MM_16_Fe_75_B_9_, and further to 10.5 kOe in MM_22_Fe_70_B_8_ containing higher RE and B concentrations.^[^ ^28^, ^29^, ^30^ ^]^ Unfortunately, the higher fraction of RE‐rich or B‐rich non‐magnetic phases sacrifices (*BH*)_max_, which shows an opposite reduction trend. Till now, a survey of the literature reveals that the highest (*BH*)_max_ is still below 15 MGOe (**Figure** **1**e), suggesting that the academic research on the MM–Fe–B system has reached a discouraging deadlock.

Accumulated research experience on the Nd–Ce–Fe–B and Nd–La–Ce–Fe–B sintered magnets has delighted us two basic strategies in improving the magnetic properties of MM–Fe–B magnets containing multiple REs, specifically, regulating the elemental segregation behaviors via deliberate alloy design during powder metallurgical processing^[^ ^31^, ^32^ ^]^ or via grain boundary diffusion processing (GBDP).^[^ ^33^, ^34^ ^]^ This considers the different phase formation disciplines of La/Ce/Pr/Nd REs when combined with Fe and other alloying elements. For example, massive Ce inevitably triggers the formation of Ce‐rich REFe_2_ phases in a large quantity,^[^ ^35^, ^36^ ^]^ while La with strong oxygen affinity easily generates the high‐melting‐point La‐rich oxide.^[^ ^37^, ^38^ ^]^ This complexity introduces intricate implications and also offers a multitude of opportunities for tailoring the intrinsic magnetic properties and extrinsic microstructure (particularly, the constituent and distribution of intergranular phases). Accordingly, to optimize the La/Ce‐rich intergranular phases with better wettability, and simultaneously to realize the higher *H*_A_ of 2:14:1 matrix phase are critical for developing high‐performance microcrystalline MM–Fe–B sintered magnets.

Minor Cu alloying has been reported to induce novel Cu‐dissolved REFe_2_ phase and Cu‐dissolved RE‐rich phase in the Nd–Ce–Fe–B sintered magnets,^[^ ^39^ ^]^ which underscores the critical functions of alloying strategy in optimizing the composition and distribution of intergranular phases. Small quantities of Co, Al, Si, and Ti alloying have also proven to enhance the coercivity of nanocrystalline MM–Fe–B magnets.^[^ ^40^, ^41^, ^42^, ^43^ ^]^ The idea of Pr_80_Ga_20_ GBDP has been successfully applied to form the anti‐ferromagnetic RE_6_Fe_13_Ga intergranular phases at triple junctions (TJs) and grain boundaries (GBs), which contribute to the magnetic isolation of 2:14:1 matrix phase and coercivity enhancement.^[^ ^44^ ^]^ For the microcrystalline MM–Fe–B sintered magnets with elemental doping strategy or GBDP strategy, the long‐term sintering, annealing or diffusion process shall exert more complicate microstructural evolution, which however, have rarely been reported to date.

Whether the MM–Fe–B system has the potential to be used as an alternative permanent material remains an intriguing question. Among versatile additives, the abundant and inexpensive Al possesses the prominent advantage in modifying the wettability of intergranular phases. However, precise control of Al doping concentration is imperative due to its high solubility in the 2:14:1 matrix phase with resultant magnetic dilution effects.^[^ ^45^, ^46^ ^]^ Excessive Al incorporation may compromise the overall magnetic performance. In this contribution, new attempts are made to thoroughly delve into the role of increasing Al addition (from 0.4 to 1.0 wt%) on the extrinsic and intrinsic performance of MM–Fe–Al–Ga–B sintered magnets. For simplicity, those sintered magnets with respective Al contents of 0.4, 0.7 and 1 wt% are designated as Al‐0.4, Al‐0.7 and Al‐1 MM–Fe–Al–Ga–B magnets in the remainder of the paper. Results show that the proposed Al‐doping strategy offers a facile route towards high‐(*BH*)_max_. The followed Nd/Pr‐based GBDP strategy further harnesses high‐*H*_cj_. Ultimately, comprehensive performance with *H*_cj_ = 10.14 kOe, *B*_r_ = 11.03 kG, and (*BH*)_max_ = 28.02 MGOe that is far superior to previously reported ones is achieved. This reveals that the resource‐saving microcrystalline MM–Fe–Al–Ga–B sintered magnet with substantially boosted magnetic performance is a promising permanent material with foreseeable future for massive application.

## 2 Results

### 2.1 Substantially Boosted Magnetic Performance

Figure 1 displays the substantially boosted magnetic performance of microcrystalline MM–Fe–Al–Ga–B sintered magnets via the proposed two‐step strategy, i.e., Al‐doping strategy towards high‐(*BH*)_max_, and subsequential Nd/Pr‐based GBDP strategy towards high‐*H*_cj_. Figure 1a,b compare the magnetic properties of the Al‐0.4, Al‐0.7 and Al‐1 MM–Fe–Al–Ga–B magnets measured at ambient temperature (20 °C). From the *J*–*H* demagnetization curves in Figure 1a, the Al‐0.7 magnet possesses simultaneously increased coercivity *H*_cj_ and remanence *B*_r_ (*H*_cj_ = 2.85 kOe, *B*_r_ = 11.03 kG), compared with the Al‐0.4 magnet (*H*_cj_ = 1.73 kOe, *B*_r_ = 10.98 kG). However, for the Al‐1 magnet with higher Al concentration, *B*_r_ dramatically reduces to 10.38 kG, with *H*_cj_ of 2.09 kOe. Visualization of maximum energy product (*BH*)_max_ values from the *B*–*H* curves in Figure 1b clearly reveals the optimal (*BH*)_max_ of 21.28 MGOe for the Al‐0.7 magnet, compared with 13.79 MGOe for the Al‐0.4 magnet and 15.41 MGOe for the Al‐1 magnet. Figure 1c,d compares the magnetic properties of the Al‐0.7 magnet before and after Nd/Pr‐based GBDP. *H*_cj_ increases remarkably from 2.85 to 10.14 kOe with simultaneously improved (*BH*)_max_ from 21.28 to 28.02 MGOe, revealing the high‐*H*_cj_ achieved via the subsequential Nd/Pr‐based GBDP. Figure 1e summarizes the benchmark (*BH*)_max_ of two representative categories of MM–Fe–B magnets from available publications with similar MM compositions (Table S1, Supporting Information). Note that our MM–Fe–Al–Ga–B magnet hits a record‐high (*BH*)_max_ of 28.02 MGOe via two‐step strategy, far beyond others in both nanocrystalline MM–Fe–B magnets (0.89–13.84 MGOe) and microcrystalline MM–Fe–B magnets (2.40–8.05 MGOe).

### 2.2 Merits of Al‐Doping Strategy

**Figure 2:**
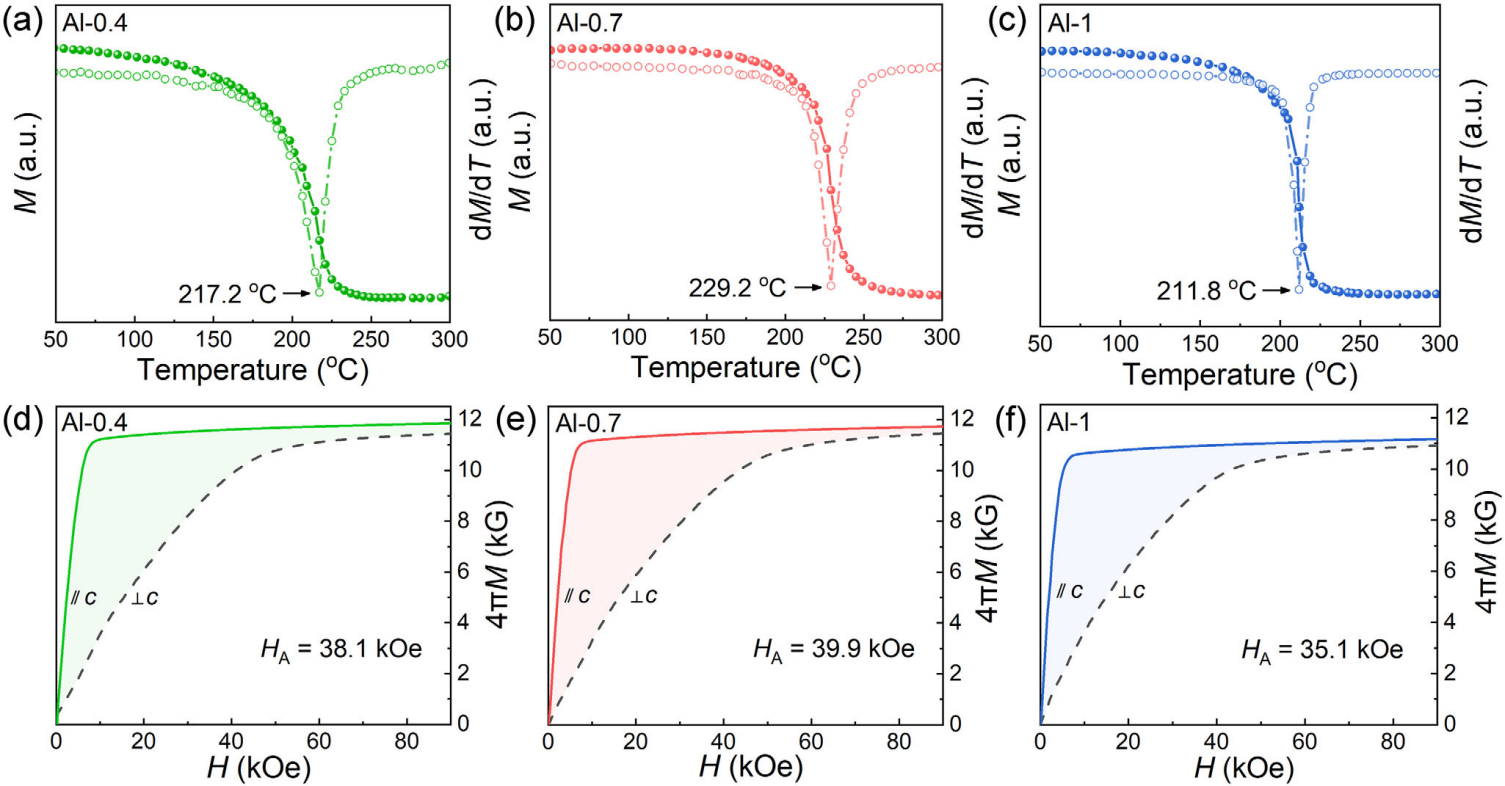
Intrinsic magnetic properties of the Al‐0.4, Al‐0.7 and Al‐1 MM–Fe–Al–Ga–B magnets. Normalized *M*–*T* and derived d*M*/d*T*–*T* curves to obtain the intrinsic Curie temperature *T*_C_ of 2:14:1 matrix phase: a) Al‐0.4, b) Al‐0.7, and c) Al‐1 magnets. Initial *M*–*H* curves along easy (∥*c* axis) and hard (⊥*c* axis) axes to obtain the intrinsic magnetocrystalline anisotropy field *H*_A_: d) Al‐0.4, e) Al‐0.7 and f) Al‐1 magnets.

To unveil the merits of Al‐doping strategy towards high‐(*BH*)_max_, both the intrinsic magnetic origin and the extrinsic microstructural contribution are analyzed in the following. **Figure** **2** compares the intrinsic magnetic properties of the Al‐0.4, Al‐0.7 and Al‐1 magnets. Figure 2a–c shows the normalized *M*–*T* and derived d*M*/d*T*–*T* curves to obtain the intrinsic Curie temperature *T*_C_ of 2:14:1 matrix phase. Similarly, through the initial *M*–*H* curves parallel and perpendicular to *c* axis of the anisotropic magnet in Figure 2d–f, the corresponding intrinsic magnetocrystalline anisotropy field *H*_A_ can be obtained. The Al‐0.7 magnet possesses the highest *T*_C_ and *H*_A_ values (*T*_C_ ≅ 229.2 °C, *H*_A_ ≅ 39.9 kOe), compared to the Al‐0.4 magnet (*T*_C_ ≅ 217.2 °C, *H*_A_ ≅ 38.1 kOe) and the Al‐1 magnet (*T*_C_ ≅ 211.8 °C, *H*_A_ ≅ 35.1 kOe). It suggests that all the intrinsic magnetic properties do not follow the linearly increasing or decreasing rule with the increasing Al concentration, while are maximized at an optimal Al doping of 0.7 wt%. Inductively coupled plasma–atomic emission spectroscopy (ICP–AES) result in Table S2 (Supporting Information) confirms the gradually increased Al doping for the Al‐0.4, Al‐0.7 and Al‐1 magnets. This also rules out the influence of deviated actual composition from the nominal one on the maximized intrinsic magnetic properties of the Al‐0.7 magnet.

**Figure 3:**
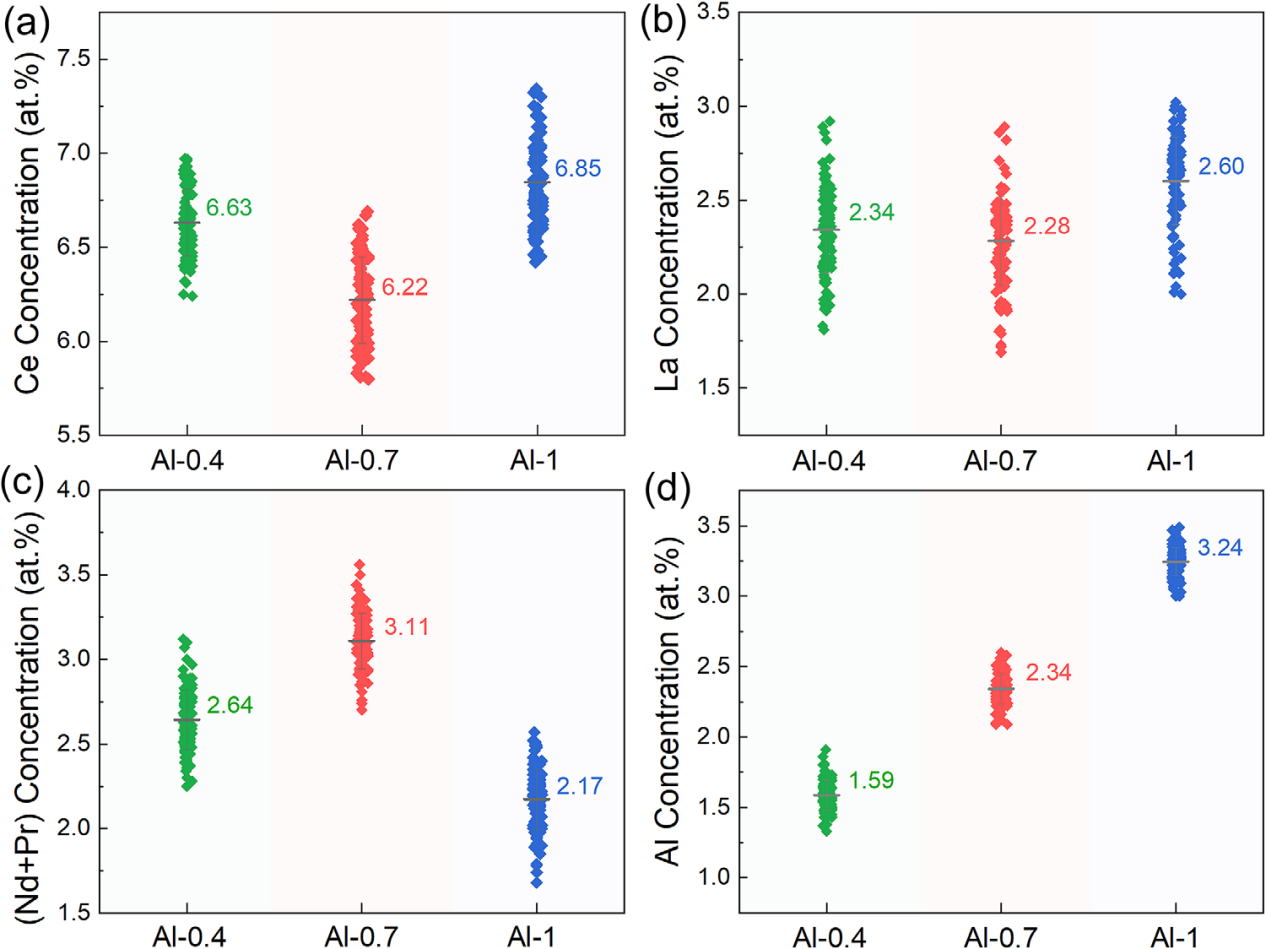
Statistical analysis of a) Ce, b) La, c) (Nd+Pr), and d) Al concentration gradients by detecting approximately 100 regions of 2:14:1 matrix phase grains for each Al‐0.4, Al‐0.7 and Al‐1 magnet.

Note that the intrinsic magnetic properties stem from the 2:14:1 matrix phase grain. To uncover the origin of intrinsic difference between the Al‐0.4, Al‐0.7 and Al‐1 magnets, statistical EDXS results on the elemental concentration of 2:14:1 matrix phase averaged ≈100 typical regions are plotted in **Figure** **3**. Figure 3a depicts that the predominant Ce concentration inside 2:14:1 matrix phase is reduced from ≈6.63 at% in the Al‐0.4 magnet to ≈6.22 at% in the Al‐0.7 magnet, while increased to ≈6.85 at% in the Al‐1 magnet. Similar tendency is also observed for the La element, as shown in Figure 3b. Conversely, the (Nd+Pr) concentration in Figure 3c is increased from ≈2.64 at% in the Al‐0.4 magnet to ≈3.11 at% in the Al‐0.7 magnet, while reduced to ≈2.17 at% in the Al‐1 magnet. Since the *H*_A_ and *T*_C_ of Ce_2_Fe_14_B and La_2_Fe_14_B is much lower than that of Nd_2_Fe_14_B and Pr_2_Fe_14_B,^[^ ^16^, ^53^ ^]^ the variations of RE concentration inside 2:14:1 matrix phase grains are consistent with the measured *H*_A_ and *T*_C_ plotted in Figure 2. In Figure 3d, the Al concentration is increased linearly from ≈1.59 at% for the Al‐0.4 magnet to ≈3.24 at% for the Al‐1 magnet, as traditionally viewed that Al tends to diffuse into the 2:14:1 matrix phase.^[^ ^46^, ^54^ ^]^

**Figure 4:**
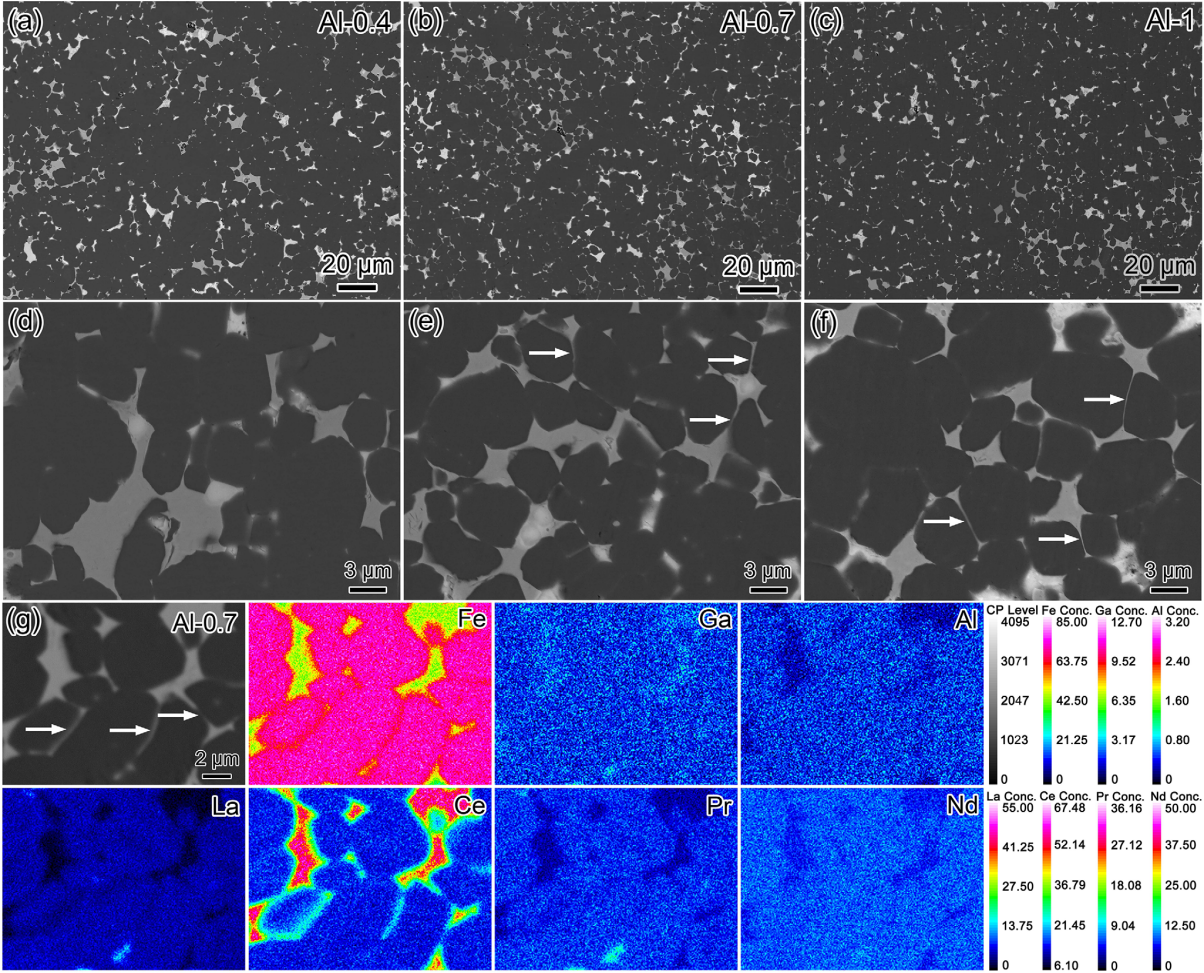
Low‐ and high‐magnification BSE SEM images of a,d) Al‐0.4, b,e) Al‐0.7 and c,f) Al‐1 magnets. g) Corresponding EPMA images of one typical region in the Al‐0.7 magnet.

Further investigation is conducted to uncover the extrinsic microstructural role governing the (*BH*)_max_ of MM–Fe–Al–Ga–B magnets. Back‐scattered electron (BSE) SEM images in **Figure** **4**a–f disclose the morphology of the Al‐0.4, Al‐0.7 and Al‐1 magnets. The dark‐grey regions refer to the RE_2_Fe_14_B matrix phase, while the light‐grey regions correspond to the intergranular phase. When comparing the Al‐0.7 and Al‐1 magnets (high Al) with the Al‐0.4 magnet (low Al), at first glance, it is found that apart from the agglomerated light‐gray TJs, more thin GBs with the similar contrast appears (as marked by the white arrows in enlarged Figure 4e,f). From the EPMA images of one typical region in the Al‐0.7 magnet (Figure 4g), both the large TJs and thin GBs are enriched with Fe and Ce, but relatively lean with La, Nd and Pr. The atomic ratio of RE to Fe in those light‐grey TJ phases is close to 1:2, being similar to REFe_2_ phase occurred in conventional Nd–Ce–Fe–B.^[^ ^55^, ^56^ ^]^ Note that these light‐gray REFe_2_ phases are aggregated in large TJs in the Al‐0.4 magnet (low Al), while tend to form continuous GBs in the Al‐0.7 and Al‐1 magnets (high Al), suggesting the crucial role of Al in regulating the phase constituents and distributions of the MM–Fe–B magnets.

**Figure 5:**
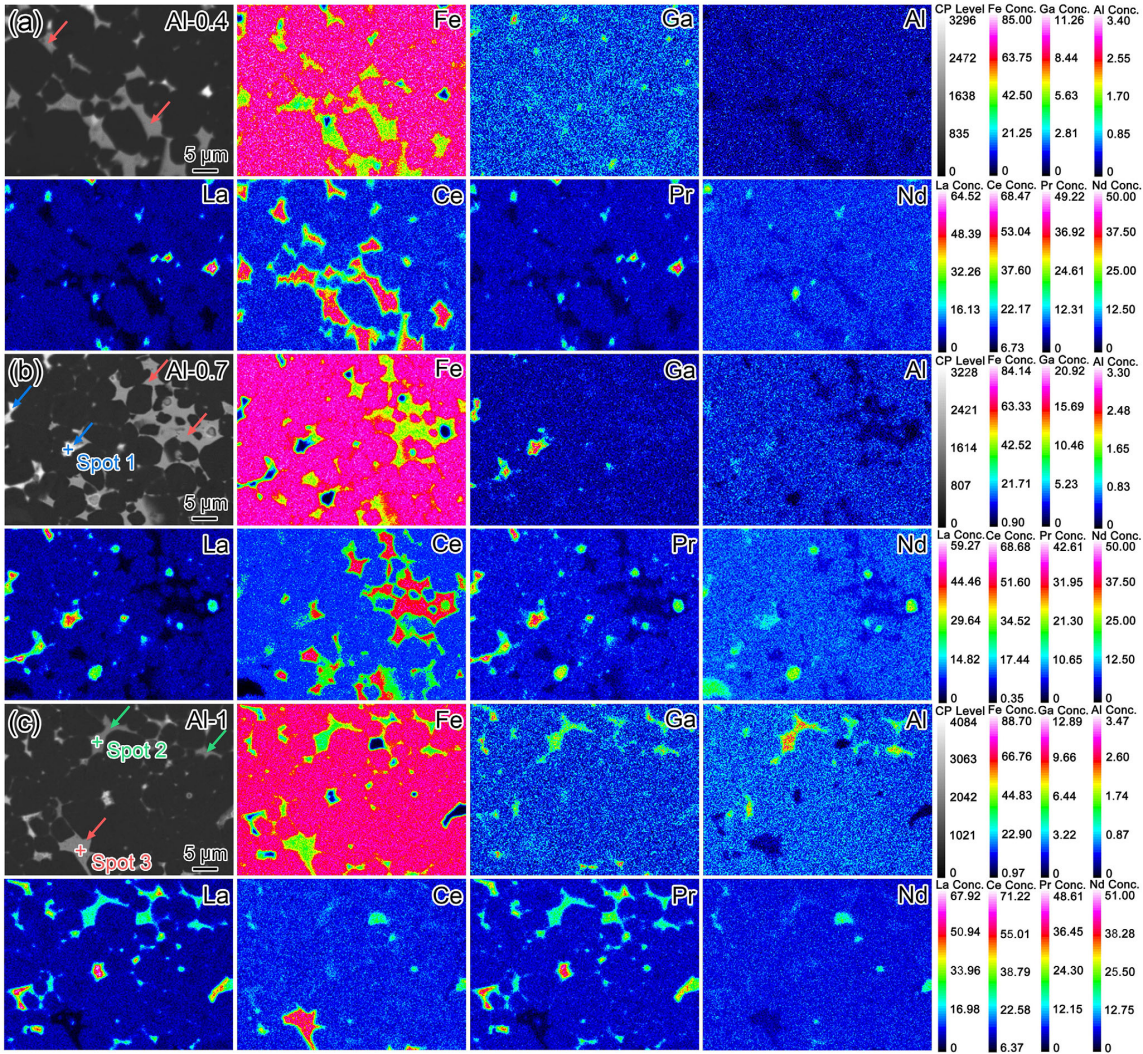
BSE SEM and corresponding EPMA images for a) Al‐0.4, b) Al‐0.7, and c) Al‐1 magnets. The red, green and blue arrows indicate the Ce‐rich REFe_2_ phases, La‐rich RE_6_(Fe, Al, Ga)_14_ phases and LaCe‐rich RE–(Al, Ga) phases, respectively.

Via high‐magnification EPMA analysis displayed in **Figure** **5**, more information on the segregated elemental distribution within the intergranular regions is obtained. Three types of intergranular phases can be identified. The first type is rich in Ce while lean in La, Nd and Pr, which corresponds to the Ce‐rich REFe_2_ intergranular phase. Such 1:2‐type intergranular phase is dominant in the Al‐0.4 and Al‐0.7 magnets, as highlighted by the red arrows in Figure 5a,b. The second type is La‐rich RE_6_(Fe, Al, Ga)_14_ intergranular phase, which is commonly visible in the Al‐1 magnet, as highlighted by the green arrows in Figure 5c. Although the Ce‐rich REFe_2_ and La‐rich RE_6_(Fe, Al, Ga)_14_ intergranular phases possess the similar Fe concentration and the same light‐grey contrast in the BSE mode, different La/Ce and Al/Ga segregation behaviors help distinguish their distribution, as verified by the elemental mappings in Figure 5c and EDXS spectra in Figure S2b,c (Supporting Information). Spot 2 with the approximate chemical composition of La_13.87_Ce_7.16_Pr_0.50_Nd_4.56_Fe_55.08_Al_12.54_Ga_4.96_Others_1.33_ (at%) corresponds to the La‐rich RE_6_(Fe, Al, Ga)_14_ intergranular phase, while Spot 3 with the approximate chemical composition of La_0.59_Ce_30.30_Pr_0.10_Nd_0.56_Fe_63.29_Al_2.29_Ga_0.53_Others_2.34_ (at%) corresponds to the Ce‐rich REFe_2_ intergranular phase. The third type is the bright‐contrast LaCe‐rich RE–(Al, Ga) phase typically observed in the Al‐0.7 magnet, as highlighted by the blue arrows in Figure 5b and verified by the EDXS spectra in Figure S2a (Supporting Information) (Spot 1, La_36.90_Ce_11.74_Pr_2.64_Nd_6.37_Fe_9.70_Al_7.73_Ga_23.05_Others_1.87_, at%). Apparently, the increasing Al doping not only witnesses the higher Al concentration dissolved in 2:14:1 matrix phase (Figure 3d), but also induces the obvious intergranular phase transformation, which subsequently influence the final magnetic performance.

**Figure 6:**
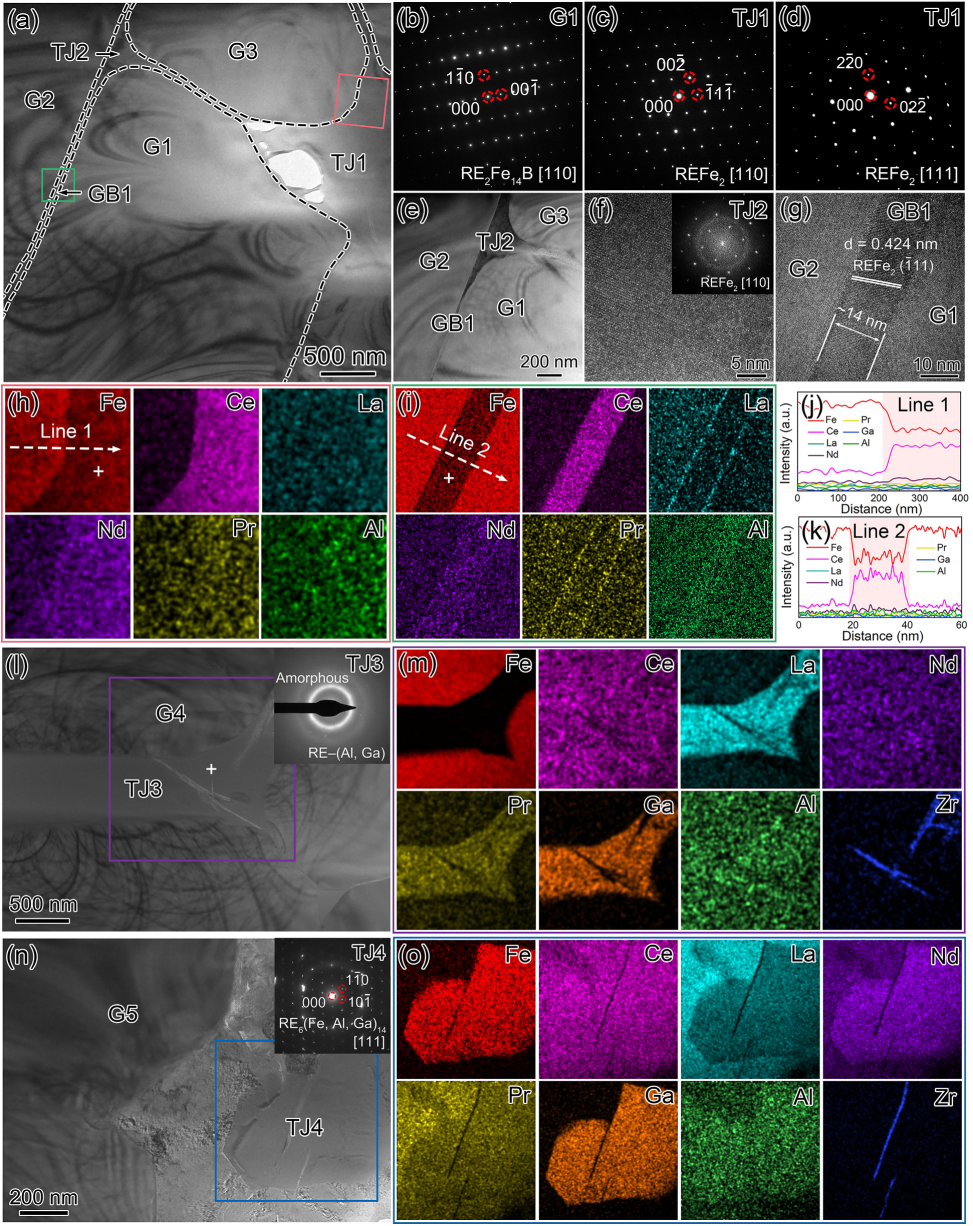
TEM analysis of three typical regions in the Al‐0.7 magnet. a) Overview BFI. The interface between RE_2_Fe_14_B matrix phase and intergranular TJ/GB phase is denoted by the dashed lines marked in black. SAED patterns of b) RE_2_Fe_14_B grain G1 along the zone axis of [110] and c,d) REFe_2_ tripe junction TJ1 along the zone axes of [110] and [111]. e) BFI of TJ2 and adjacent grain boundary GB1 with the thickness of ≈14 nm. HRTEM images of f) TJ2 and g) continuous GB1, the upper‐right inset in (f) shows the FFT pattern of REFe_2_ TJ2. Corresponding STEM‐EDXS mappings of Fe, Ce, La, Nd, Pr and Al for h) the G3/TJ1 interface marked with red square and i) the G2/GB1/G1 interface marked with green square shown in (a). The symbol “+” indicates the positions of the EDXS spectra provided in Figure S3 (Supporting Information). Scan profiles along j) Line 1 shown in (h), and k) Line 2 shown in (i), respectively. l) BFI with the SAED pattern of LaCe‐rich RE–(Al, Ga) TJ3 in the upper‐right inset. m) Corresponding STEM‐EDXS mappings acquired from the purple square region shown in (l). n) BFI with the SAED pattern of La‐rich RE_6_(Fe, Al, Ga)_14_ TJ4 in the upper‐right inset. o) Corresponding STEM‐EDXS mappings acquired from the blue square region shown in (n).

Further TEM analysis on the intergranular phases typically observed in the Al‐0.7 magnet is shown in **Figure** **6**. Figure 6a provides the overview BFI of the first typical region. Figure 6b–d displays the SAED patterns of tetragonal RE_2_Fe_14_B grain G1 (along the zone axis of [110]), and cubic REFe_2_ TJ1 (along the zone axes of [110] and [111]). Figure 6e provides the BFI of TJ2 and adjacent GB1. According to the HRTEM images in Figure 6f,g, both the agglomerated TJ2 and the continuous GB1 with a thickness of ≈14 nm can be indexed as Ce‐rich REFe_2_ phase, suggesting the improved wettability of these REFe_2_ phases to infiltrate into the interface of adjacent RE_2_Fe_14_B grains. The STEM‐EDXS mappings and corresponding line scan profiles in Figure 6h–k reveal that the intergranular TJ1 and GB1 are depleted in La, Nd and Pr, while rich in Ce and Fe, being consistent with the characteristic composition of Ce‐rich REFe_2_ phase. According to the EDXS spectra provided in Figure S3a,b (Supporting Information), the measured Al concentration is ≈4.07 at% within GB1, much higher than that of ≈2.20 at% within TJ1, further suggesting the crucial role of Al in improving the wettability of Ce‐rich REFe_2_ GB phase.

Figure 6l showcases the overview BFI of the second typical region. STEM‐EDXS mappings in Figure 6m and supplementary EDXS spectrum in Figure S3c (Supporting Information) reveal that TJ3 is rich in RE (mainly La and Ce) and non‐RE (Al, Ga), with the measured composition of La_34.50_Ce_11.20_Pr_3.65_Nd_8.50_Al_7.47_Ga_25.43_Fe_4.14_Others_5.11_ (at%), corresponding to the LaCe‐rich RE–(Al, Ga) phase. For the third typical region shown in Figure 6n,o, the La‐rich tetragonal RE_6_(Fe, Al, Ga)_14_ TJ4 along the zone axis of [111] can be identified. Above results collectively verify the accelerated La/Ce segregation and resultant formation of LaCe‐rich RE–(Al, Ga), La‐rich RE_6_(Fe, Al, Ga)_14_, and Ce‐rich REFe_2_ intergranular phases with better wettability.

**Figure 7:**
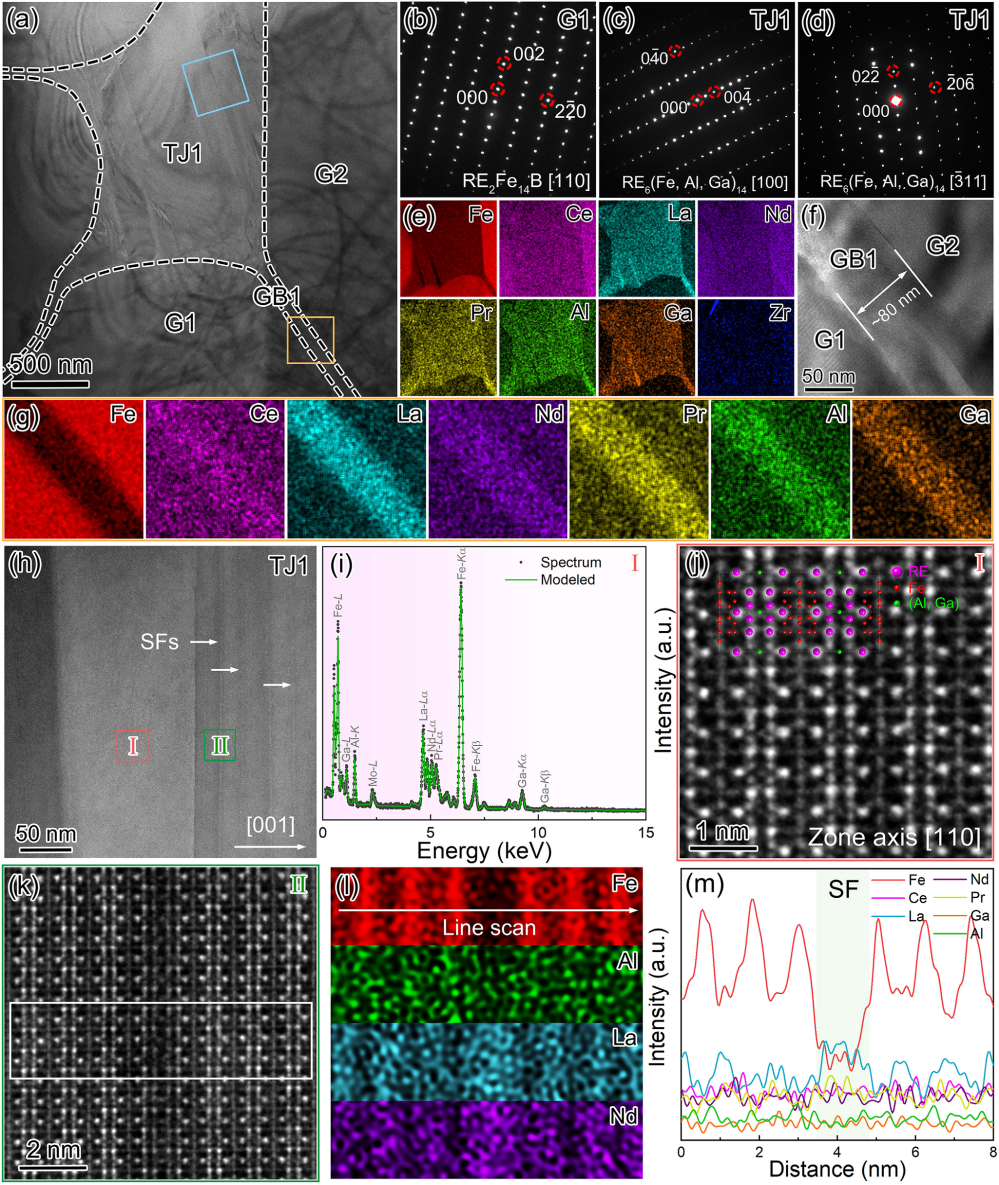
TEM analysis of one typical region in the Al‐1 magnet. a) Overview BFI. b) SAED pattern of RE_2_Fe_14_B grain G1. SAED patterns of La‐rich RE_6_(Fe, Al, Ga)_14_ TJ1 along zone axes of c) [100] and d) [$\bar{3}$11]. e) Elemental distribution mappings. f) Enlarged BFI of the G1/GB1/G2 interface, and g) corresponding elemental distribution mappings. h) HAADF‐STEM image taken from the cyan‐square region in (a). i) STEM‐EDXS spectrum and j) atomic‐scale HAADF‐STEM image acquired from the red‐square region I in (h). k) Atomic‐scale HAADF‐STEM image of the green‐square region II in (h) with obvious SFs. (l) STEM‐EDXS elemental mappings of the white‐rectangular region in (k), m) corresponding line scan profiles.

To explore the intergranular phase transformation upon higher Al doping, **Figure** **7**a provides the overview BFI of one typical region in the Al‐1 magnet. From the SAED patterns in Figure 7b–d, G1 refers to the tetragonal RE_2_Fe_14_B phase along the [110] zone axis, and TJ1 refers to the tetragonal RE_6_(Fe, Al, Ga)_14_ phase along the [100] and [$\bar{3}$11] zone axes. Corresponding STEM‐EDXS mappings in Figure 7e,g show the enrichment of Fe, RE (mainly La) and non‐RE (Al and Ga) in the intergranular TJ1 and continuous GB1 with a thickness of ≈80 nm, which accord well with the stoichiometry of La‐rich RE_6_(Fe, Al, Ga)_14_ phase.

Further atomic‐scale STEM analysis is performed focusing on the La‐rich RE_6_(Fe, Al, Ga)_14_ TJ1 with lamellar distribution (Figure 7h). Obvious structural defects of stacking faults (SFs) are gathered inside the right lamella (region II marked with the white arrows). Comparably, for region I in close proximity to region II, the STEM‐EDXS spectrum in Figure 7i and atomic‐scale HAADF‐STEM image in Figure 7j confirm the formation of La‐rich RE_6_(Fe, Al, Ga)_14_ phase, with measured near‐stoichiometric composition of La_11.96_Ce_5.40_Pr_2.58_Nd_3.11_Fe_59.60_Al_7.41_Ga_4.74_Others_5.20_ (at%). The structure model of RE_6_(Fe, Al, Ga)_14_ unit cell along the [110] zone axis is overlaid on the experimental atomic‐scale HAADF‐STEM image, showing a good correspondence. Figure 7k shows an enlarged image of region II, revealing the presence of obvious SF in the middle of the image. Corresponding STEM‐EDXS mappings and line scan profiles in Figure 7l,m reveal that the SF is depleted in Fe, Al and Ga, while rich in La, Ce, Pr and Nd. In fact, the SFs are commonly observed in those La‐rich RE_6_(Fe, Al, Ga)_14_ intergranular phases, as further confirmed by Figure S4 (Supporting Information). Being different from the previously reported 6:14‐type phase,^[^ ^57^, ^58^ ^]^ this is the first time to display the existence of SFs in high Al containing 6:14 phases. It is also noted that there still exists massive directly contacted matrix phase grains without the presence of continuous GBs, as displayed at the G1/GB1/G2 interface in Figure S5 (Supporting Information), suggesting the necessity of additional technology with modified microstructural characteristic towards high‐*H*_cj_ MM–Fe–B magnet.

### 2.3 Merits of Nd/Pr‐Based GBDP Strategy

**Figure 8:**
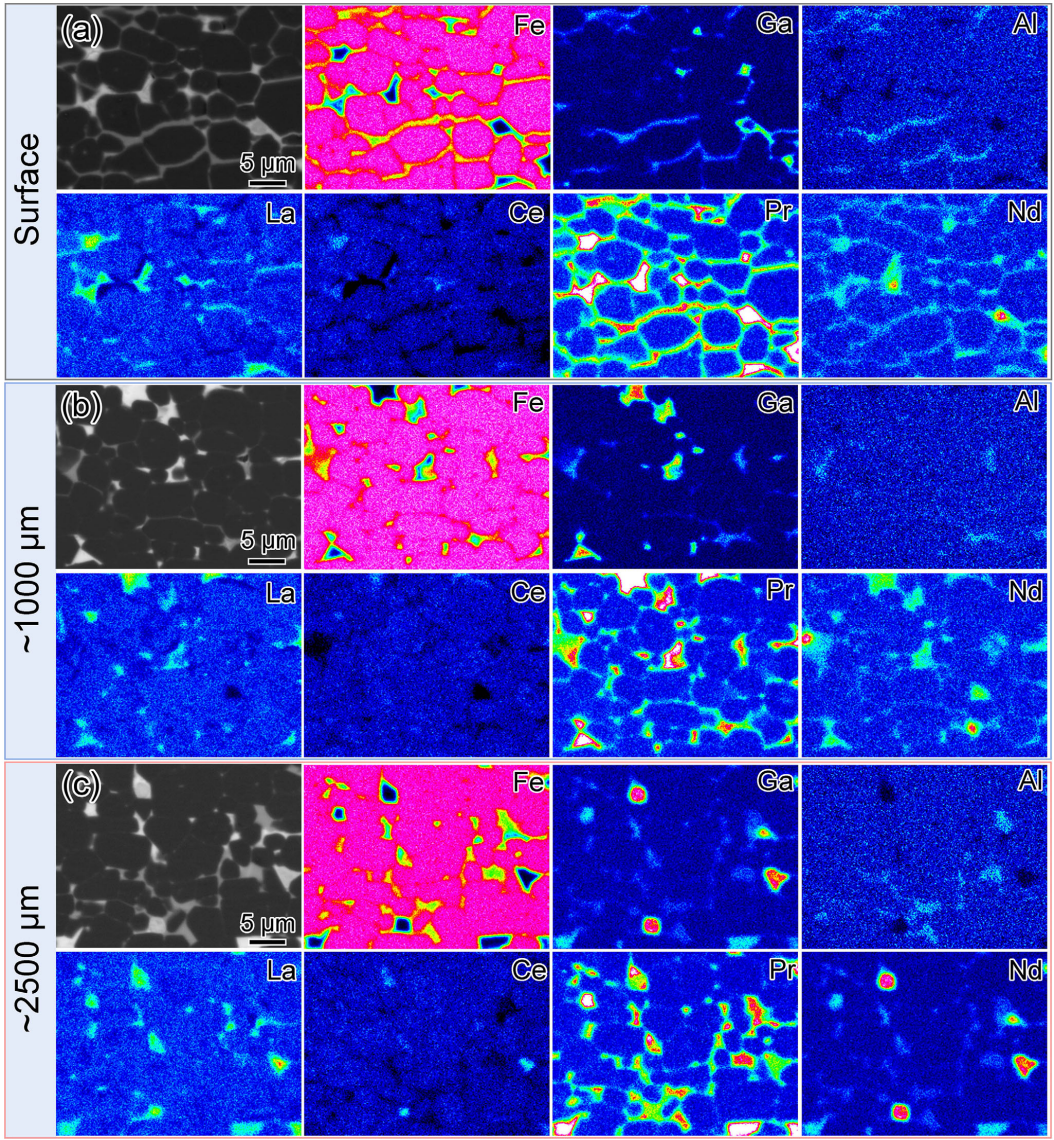
BSE SEM and EPMA images of the Nd/Pr‐based GBDP MM–Fe–Al–Ga–B magnet: a) near the capping surface, at increasing diffusion depths of b) ≈1000 µm and c) ≈2500 µm. Note that the elemental concentration bars are adjusted within the same range (0–80 wt% Fe, 0–20 wt% Ga, 0–3 wt% Al, 0–34 wt% La, 10–58 wt% Ce, 0–40 wt% Pr and 0–40 wt% Nd).

Based on the aforementioned Al‐doping strategy to achieve a (*BH*)_max_ of 21.28 MGOe, we further perform the Nd/Pr‐based GBDP strategy to boost an enormous enhancement of *H*_cj_ from 2.85 to 10.14 kOe in the Al‐0.7 magnet. To gain more details about the microstructural evolution, the BSE SEM and EPMA images from surface to the center (≈2500 µm) of the Nd/Pr‐based GBDP magnet are provided in **Figure** **8**. The formation of well‐constructed Nd/Pr‐rich shell surrounding La/Ce‐rich matrix grains can be clearly observed even at a diffusion depth of ≈2500 µm (Figure 8c), which shall enhance the local *H*_A_. More significantly, the light‐grey intergranular phases form in agglomerated TJs and continuous GBs throughout the bulk magnet. Combined with the EDXS analysis in Table S3 (Supporting Information), those intergranular TJs and GBs are rich in Fe, RE (mainly Pr and Nd) and non‐RE (Al and Ga) with the atomic ratio of REs to (Fe+Al+Ga) close to 6:14, revealing the dominance of Nd/Pr‐rich RE_6_(Fe, Al, Ga)_14_ phase in the Nd/Pr‐based GBDP magnet.

**Figure 9:**
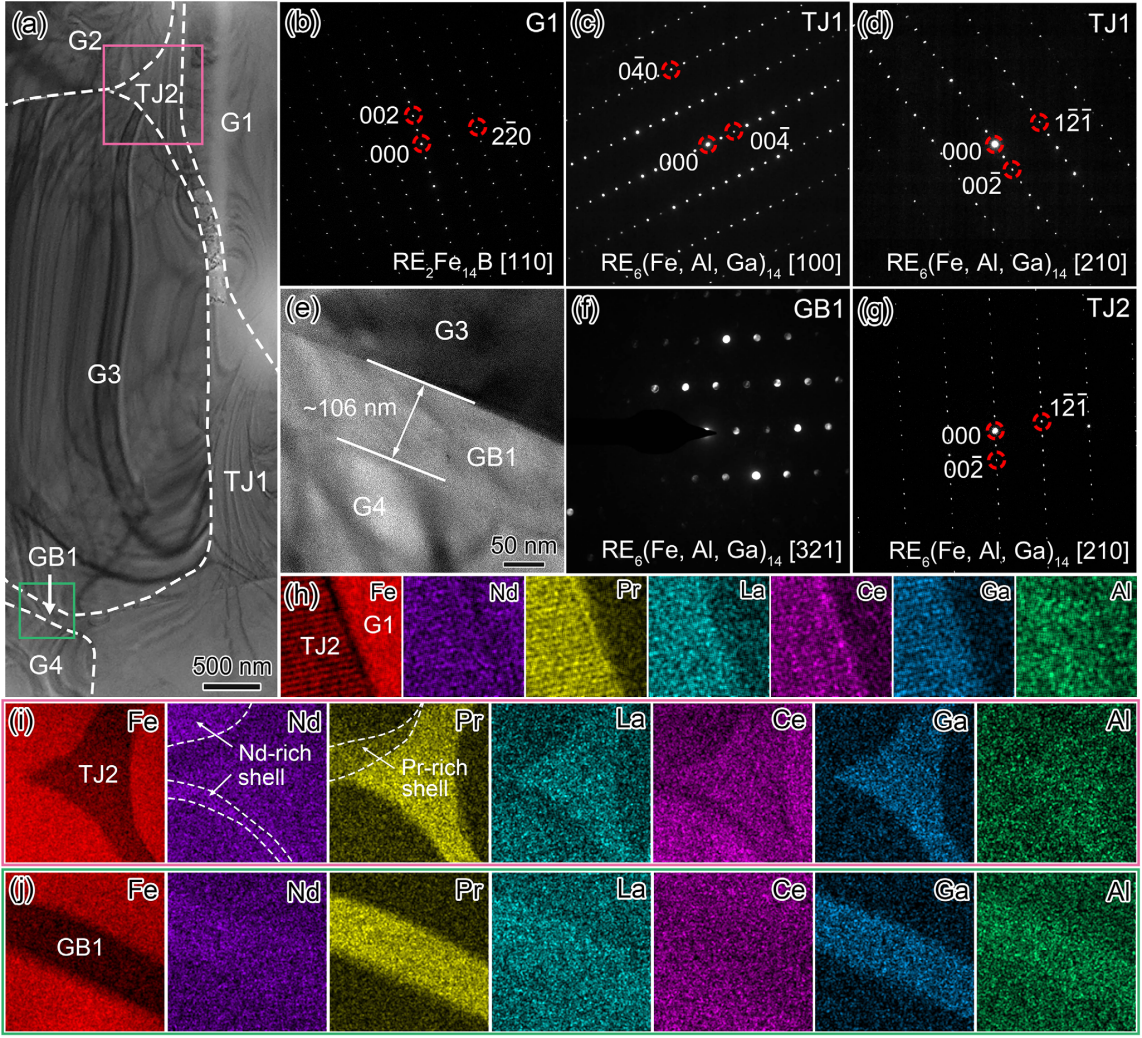
TEM analysis of one typical region in the Nd/Pr‐based GBDP MM–Fe–Al–Ga–B magnet. a) Overview BFI. b) SAED pattern of RE_2_Fe_14_B grain G1. SAED patterns of RE_6_(Fe, Al, Ga)_14_ TJ1 along zone axes of c) [100] and d) [210]. e) Enlarged BFI of the G3/GB1/G4 interface, showing the continuous GB1 layer with a thickness of ≈106 nm. f) NBED pattern of RE_6_(Fe, Al, Ga)_14_ GB1 along zone axis of [321]. g) SAED pattern of RE_6_(Fe, Al, Ga)_14_ TJ2 along zone axis of [210]. Elemental distribution mappings of h) the TJ2/G1 interface, i) enlarged TJ2 region and j) the G3/GB1/G4 interface.

Further BFI, SAED and NBED patterns in **Figure** **9**a–g verify the formation of NdPr‐rich RE_6_(Fe, Al, Ga)_14_ TJ1, TJ2 and GB1 isolating the neighboring matrix phase grains G1/G2/G3/G4. Together with the STEM‐EDXS mappings in Figure 9i, the Nd/Pr‐rich magnetically hardening shells surrounding the La/Ce‐rich RE_2_Fe_14_B matrix phase grains can be clearly observed, as marked with the white arrows. Above microstructural characteristics shall further enhance the local *H*_A_ to inhibit the nucleation process and impede the rapid propagation of demagnetization domain.

**Figure 10:**
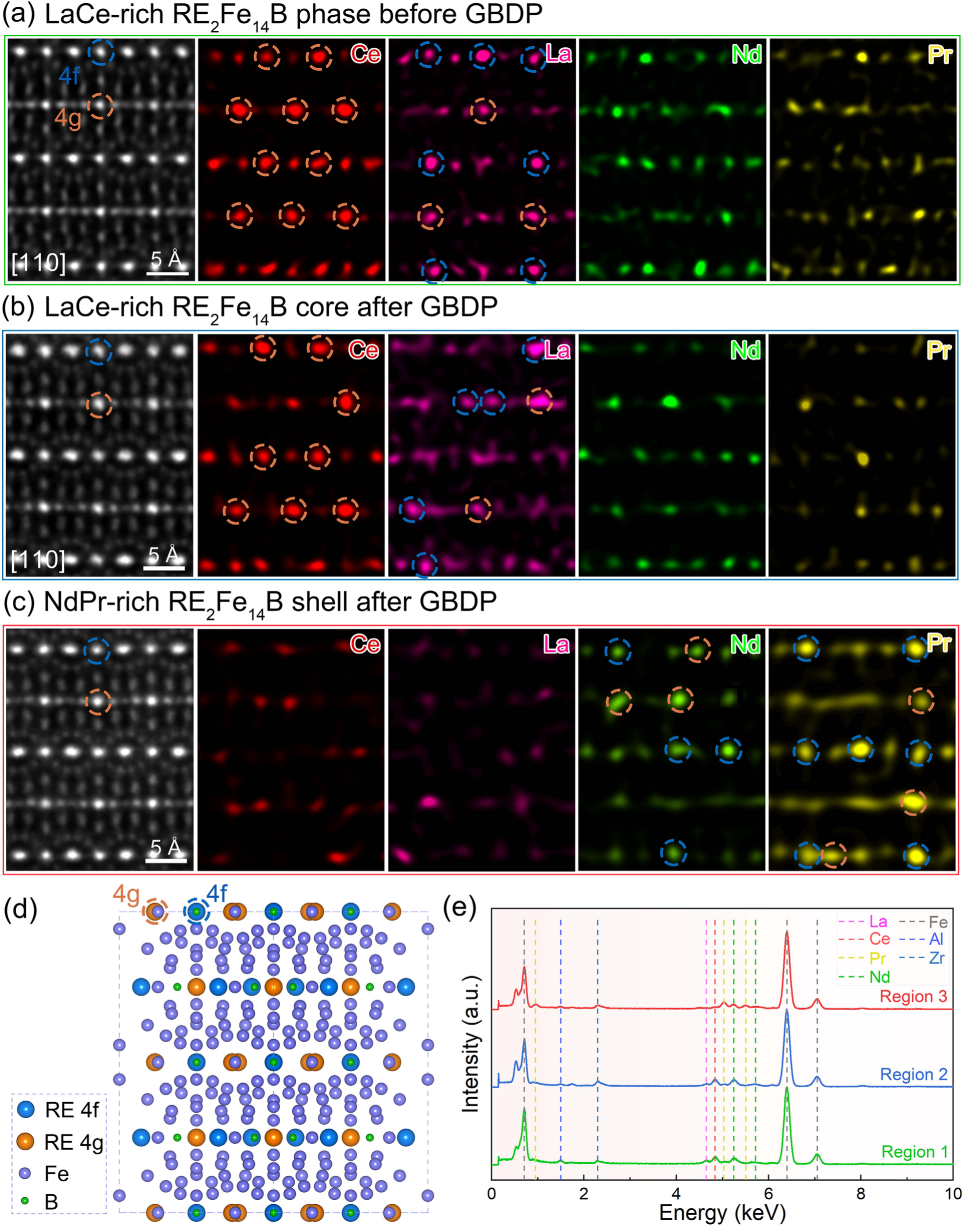
Site occupation of REs within 2:14:1 matrix phase in the MM–Fe–Al–Ga–B magnet before and after Nd/Pr‐based GBDP. HAADF image of atomic structure recorded along the [110] zone axis, and corresponding EDXS mappings for individual RE elements of Ce, La, Nd and Pr: a) LaCe‐rich RE_2_Fe_14_B phase before GBDP, b) LaCe‐rich RE_2_Fe_14_B core and c) NdPr‐rich RE_2_Fe_14_B shell after GBDP. d) Schematic illustration of RE_2_Fe_14_B crystal structure containing two non‐equivalent crystallographic 4f and 4g RE positions. e) EDXS spectra taken from Region 1 in (a), Region 2 in (b) and Region 3 in (c).

To obtain atomic‐scale structural information, the lattice images of typical RE_2_Fe_14_B matrix phase and corresponding EDXS mappings in the MM–Fe–Al–Ga–B magnet before and after Nd/Pr‐based GBDP are captured using high‐resolution STEM in **Figure** **10**a–c. By examining the atomic‐resolution EDXS mappings in detail, certain site occupation of REs within the 2:14:1 lattice can be identified. For each specific element such as Ce, the brightness of an individual spot increases approximately with the number of Ce atoms in the atomic column along [110] zone axis and reveals the local Ce density. Obviously, the Ce atomic mappings with relatively higher brightness (marked with orange circles in Figure 10a,b) mainly locate at the crystallographic 4g sites both in the LaCe‐rich RE_2_Fe_14_B phase before GBDP and LaCe‐rich RE_2_Fe_14_B core after GBDP, based on the RE_2_Fe_14_B crystal structure in Figure 10d. This reveals the site preference of Ce at the larger 4g site in the MM–Fe–B magnet, similar to that reported in the Nd–Ce–Fe–B magnet.^[^ ^59^, ^60^ ^]^ Conversely, more La atoms occupy the 4f site (marked with blue circles) than 4g site (marked with orange circles) from La mappings in Figure 10a. In the NdPr‐rich RE_2_Fe_14_B shell after GBDP (Figure 10c), the overall Ce and La atomic fraction is reduced. This is understandable since more Nd and Pr atoms substitute Ce and La within the 2:14:1 lattice, as evidenced by the altered intensity of REs. Specifically, the increased Nd atoms are uniformly located at 4f and 4g sites, while Pr atoms are more likely to enter the 4f site. Corresponding EDXS spectra in Figure 10e also confirm that more pronounced Pr and Nd characteristic peaks are observed in the NdPr‐rich RE_2_Fe_14_B shell after GBDP (Region 3), compared to the LaCe‐rich RE_2_Fe_14_B phase before GBDP (Region 1) and LaCe‐rich RE_2_Fe_14_B core after GBDP (Region 2).

## 3 Discussion

**Figure 11:**
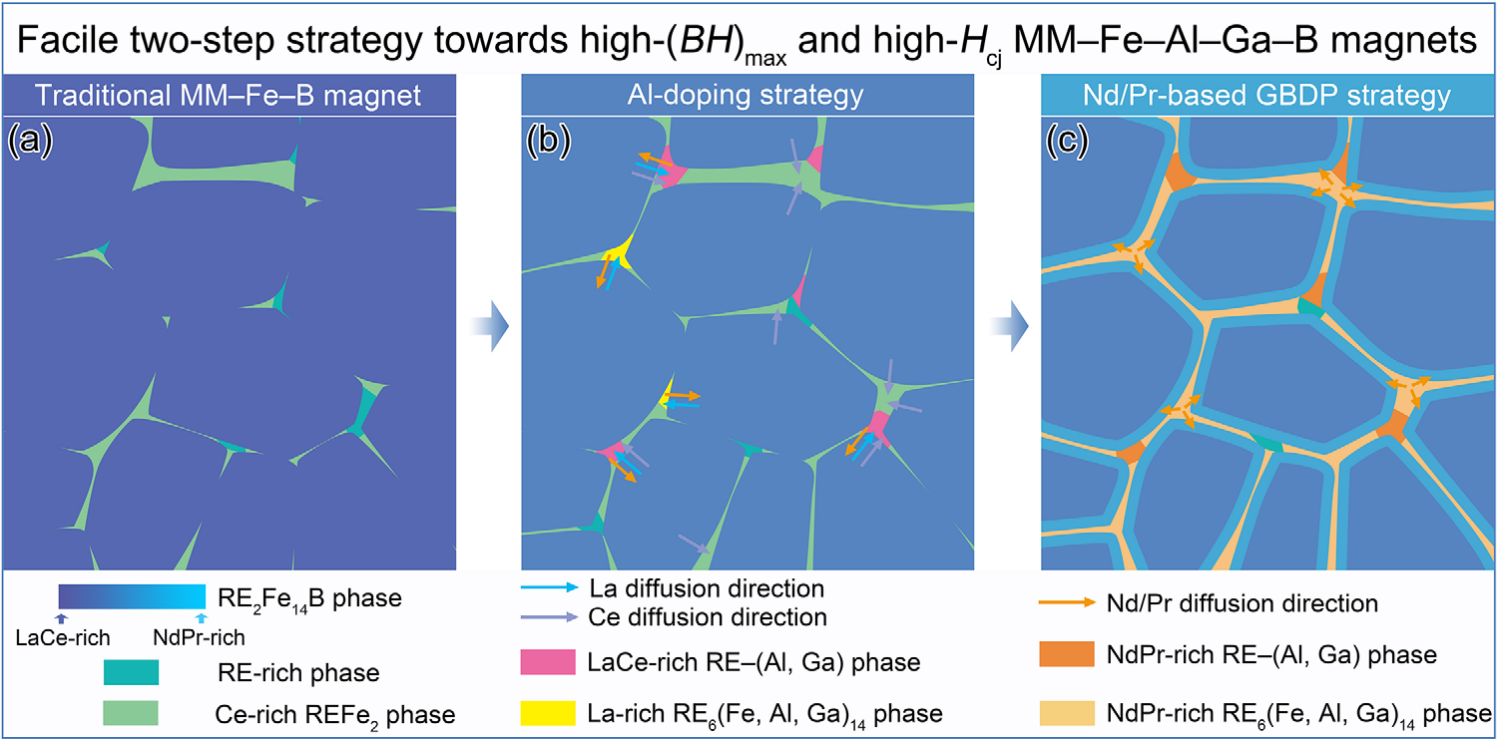
Schematic diagram of the microstructural evolutions: a) Traditional MM–Fe–B magnet, b) appropriate Al‐doping strategy towards high‐(*BH*)_max_ and c) subsequential Nd/Pr‐based GBDP strategy towards high‐*H*_cj_ MM–Fe–Al–Ga–B magnets. Different La/Ce/Pr/Nd segregation behaviors in typical intergranular phases are highlighted by arrows.

Focusing on the microcrystalline MM–Fe–B sintered magnets with resource‐saving and cost‐efficient merits, but with the long‐standing demerit of extremely‐low magnetic performance, here we propose a facile two‐step strategy towards high‐(*BH*)_max_ and high‐*H*_cj_ MM–Fe–Al–Ga–B magnets during the powder metallurgy method. Based on the multi‐scale microstructural characterization described above, we set out the schematic diagrams of microstructural evolution, as displayed in **Figure** **11**.

For traditional MM–Fe–B magnet, a trace amount of Al (0.1–0.3 wt%) is usually incorporated.^[^ ^20^, ^26^, ^61^ ^]^ Therefore a supplementary Al‐0.2 sintered magnet (0.2 wt% Al) was also prepared for comparative analysis. As illustrated in Figure S6a–d (Supporting Information), the Al‐0.2 magnet exhibits basically similar extrinsic and intrinsic magnetic performance [(*BH*)_max_ = 12.62 MGOe, *T*_C_ ≅ 218.8 °C, *H*_A_ ≅ 37.8 kOe] to the Al‐0.4 one. Corresponding BSE SEM image and EDXS elemental mappings (Figure S6e,f, Supporting Information) also reveal similar microstructural features that are dominated by the agglomerated Ce‐rich REFe_2_ TJs without identification of clear GBs. This indicates that the traditional MM–Fe–B magnet within the low‐Al doping range can be represented by the Al‐0.2 and Al‐0.4 magnets in present work, as a starting point schematically shown in Figure 11a.

Comparably, the first step of appropriate Al‐doping strategy yields a superb (*BH*)_max_ of 21.28 MGOe in the Al‐0.7 MM–Fe–Al–Ga–B magnet (Figure 1a,b), which is principally ascribed to two aspects (as depicted in Figure 11b). From the intrinsic viewpoint, the Al‐0.7 magnet possesses higher intrinsic magnetic properties than those of the Al‐0.4 and Al‐1 magnets (Figure 2). Specifically, *T*_C_ of 2:14:1 matrix phase peaks at ≈229.2 °C, and *H*_A_ maximizes at ≈39.9 kOe for the Al‐0.7 magnet, which offer ample room for extrinsic microstructural modulation. Such higher intrinsic magnetic properties are ascribed to the lower La and Ce concentrations within 2:14:1 matrix phase, i.e., Ce/La concentrations of 6.22/2.28 at% for the Al‐0.7 magnet compared to 6.63/2.34 at% for the Al‐0.4 magnet and 6.85/2.60 at% for the Al‐1 magnet according to the statistical results in Figure 3. Since the intrinsic magnetism of Nd_2_Fe_14_B/Pr_2_Fe_14_B compounds are superior to La_2_Fe_14_B/Ce_2_Fe_14_B compounds,^[^ ^16^ ^]^ more infiltration of Nd/Pr into the 2:14:1 matrix phase is a crucial intrinsic factor accounting for the remarkable (*BH*)_max_ of the Al‐0.7 magnet. Unfortunately, since the substitution of Al for Fe inevitably reduces the magnetic moment of the MM–Fe–B magnet,^[^ ^62^, ^63^ ^]^ the increasing Al dissolution inside the 2:14:1 matrix phase from 1.59 at% for the Al‐0.4 magnet to 3.24 at% for the Al‐1 magnet explains the correspondingly minimal *M*_r_ of 10.38 kG in the Al‐1 magnet upon excessive Al doping (Figure 3d).

From the extrinsic viewpoint, appropriate Al‐doping into the MM–Fe–Al–Ga–B magnets also holds significant implications for accelerating the La/Ce segregation and intergranular phase transformation. The formation of LaCe‐rich RE–(Al, Ga) phase (Figures 5b and 6l), La‐rich RE_6_(Fe, Al, Ga)_14_ phase (Figures 6n and 7), and Ce‐rich REFe_2_ phase (Figure 6a–k) with better interfacial wettability can be clearly identified. The occurrence of more continuous GBs is a general feature of the Al‐0.7 and Al‐1 magnets (high Al) compared to that of the Al‐0.4 magnet (low Al), as revealed by the SEM and EMPA images (Figures 4 and 5). The improved wettability of Ce‐rich REFe_2_ phase is presumably attributed to the well‐constructed GBs surrounding the 2:14:1 matrix phase. As verified by the statistical analysis in Figure S7 (Supporting Information), a higher Al concentration is dissolved in the REFe_2_ intergranular phase with increasing Al doping, yielding its functional role in modifying the interfacial wettability between REFe_2_ intergranular phase and RE_2_Fe_14_B matrix phase. To confirm this, a series of homogeneous alloys with nominal composition of Nd_0.25_Ce_0.75_(Fe_1‐_ *_x_* Al*_x_*)_2_ (*x* = 0, 0.0625, 0.125 and 0.25, at%) were prepared. Results show that single‐phase RE(Fe, Al)_2_ is obtained after long‐term homogenous annealing, and the melting point (*T*_m_) of RE(Fe, Al)_2_ phases decrease gradually from *T*_m1_ = 870.9 °C for *x* = 0 to *T*_m4_ = 814.3 °C for *x* = 0.25 (Figure S8, Supporting Information), indicating the Al dissolution lowers the *T*_m_ of RE(Fe, Al)_2_ phases. Subsequently, the interfacial wettability between Ce‐rich REFe_2_ intergranular phase and RE_2_Fe_14_B matrix phase is improved, which promotes the formation of more continuous REFe_2_ GBs. This microstructural evolution is also corroborated by the increased areal fraction of REFe_2_ phase from ≈3.6% in the Al‐0.4 magnet to ≈4.2% in the Al‐0.7 magnet (Figures S9 and S10, Supporting Information).

In addition to the Ce‐rich REFe₂ phase exhibiting improved interfacial wettability, the novel LaCe‐rich RE–(Al, Ga) intergranular phase with a certain areal fraction of ≈1.2% also occurs in the Al‐0.7 magnet (Figures 5b and 6l,m and Figures S9 and S10, Supporting Information). Compositional analysis in Figure S11 (Supporting Information) reveals elevated La (36.90 at%) and Ce (11.74 at%) concentrations in this LaCe‐rich RE–(Al, Ga) phase, which differs from the reported Nd/Pr‐dominated RE–Ga intergranular phase typically observed in Ga‐doped Nd–Fe–B sintered magnet.^[^ ^64^, ^65^ ^]^ Note that the formation of such LaCe‐rich RE–(Al, Ga) and more Ce‐rich REFe_2_ intergranular phases consume large quantities of La and Ce. Enormous elemental immigration occurs spontaneously at the interface of the liquid‐state intergranular phase and solid‐state 2:14:1 matrix phase, accelerating the La/Ce segregation into the intergranular regions, as visually represented by the blue and purple arrows in Figure 11b. Therefore the La/Ce concentration within the matrix phase is decreased, while the relative Nd/Pr concentration is correspondingly increased compared to the Al‐0.4 magnet (Figure 3). For the Al‐1 magnet with excessive Al doping, the LaCe‐rich RE–(Al, Ga) phase disappears, which is replaced by the La‐rich RE_6_(Fe, Al, Ga)_14_ phase with an areal fraction of ≈1.1% (Figures S9 and S10, Supporting Information). Simultaneously, the areal fraction of Ce‐rich REFe_2_ phase decreases from ≈4.2% for the Al‐0.7 magnet to ≈2.3% for the Al‐1 magnet, which is further verified by the step‐scanned XRD analysis in Figure S12 (Supporting Information). Above results indicate the phase transformation from the LaCe‐rich RE–(Al, Ga) phase and Ce‐rich REFe_2_ phase to the La‐rich RE_6_(Fe, Al, Ga)_14_ phase in consistence with previous reports in the Ga/Al/Cu containing RE–Fe–B magnets.^[^ ^44^, ^64^, ^66^ ^]^ Combined with the comparative results in Figure S11 (Supporting Information), the La and Ce concentrations within the RE_6_(Fe, Al, Ga)_14_ phase are much lower compared to the RE–(Al, Ga) and REFe_2_ intergranular phases. Therefore such a phase transformation shall release more La/Ce atoms from the intergranular regions, which in turn infiltrate into the matrix phase in the Al‐1 magnet. This accounts for the lowest La and Ce concentrations within 2:14:1 matrix phase (Figure 3) and the highest intrinsic magnetic properties (Figure 2) in the Al‐0.7 magnet.

As for the second step of Nd/Pr‐based GBDP strategy, we achieved record‐high magnetic performance with *H*_cj_ = 10.14 kOe, *B*_r_ = 11.03 kG, and (*BH*)_max_ = 28.02 MGOe (Figure 1c,d, and Figure S1, (Supporting Information)). The coercivity of 10.14 kOe exceeds the previously reported values for microcrystalline MM–Fe–B magnets (0.46–1.41 kOe). Such a high value using Nd/Pr‐based diffusion source also outperforms the reported 6.81 kOe using the heavy rare earth Tb‐based diffusion source (TbH_*x*_),^[^ ^23^ ^]^ further substantiating the advancements of the present work. Compared with the nanocrystalline magnets (notable advantage of high *H*_cj_ in‐between 0.93–10.02 kOe, with simultaneous (*BH*)_max_ in‐between 0.89–13.84 MGOe), the present MM–Fe–Al–Ga–B magnet possesses a high synergy of comparable *H*_cj_ and superior (*BH*)_max_. Collectively, these findings underscore the promising commercial potential of the present microcrystalline MM–Fe–Al–Ga–B sintered magnet.

The following Nd/Pr‐based GBDP strategy primarily induced two distinct microstructural features (as depicted in Figure 11c). Large quantities of NdPr‐rich RE_6_(Fe, Al, Ga)_14_ phases in both forms of TJs and GBs are generated. Simultaneously, the formation of NdPr‐rich magnetically hardening shell surrounding LaCe‐rich matrix grains, which are observed at an exceptionally long diffusion depth of ≈2500 µm. Elemental site occupation analysis in Figure 10 shows that within the NdPr‐rich shell, Nd and Pr atoms substitute for different crystallographic sites in the 2:14:1 crystal, i.e., Nd atoms are uniformly distributed at 4f and 4g sites, while Pr atoms tend to substitute preferentially at 4f site. Above results demonstrate that Al‐doping strategy with subsequential Nd/Pr‐based GBDP empowers us to customize the microstructure and to harvest the boosted magnetic performance in MM–Fe–Al–Ga–B sintered magnets. They also contribute to an atomic‐level understanding of the elemental segregation behaviors, which herald new possibilities for designing high‐performance Mischmetal‐based permanent material.

## 4 Conclusions

In summary, we have successfully achieved substantially boosted magnetic performance in the microcrystalline MM–Fe–Al–Ga–B permanent material via a facile two‐step strategy of Al‐doping and subsequential Nd/Pr‐based GBDP. The main conclusions can be drawn as follows: 1) Appropriate Al‐doping promotes the La/Ce segregation to the intergranular region to form massive LaCe‐rich RE–(Al, Ga) phase, Ce‐rich REFe_2_ phase, and La‐rich RE_6_(Fe, Al, Ga)_14_ phase, lowers the La/Ce concentration within the RE_2_Fe_14_B matrix phase and subsequently results in the enhanced intrinsic magnetism for the MM–Fe–Al–Ga–B magnet. 2) More Al dissolved in those Ce‐rich REFe_2_ intergranular phases shall improve its interfacial wettability and contribute to the formation of continuous REFe_2_ GBs. While excessive Al dissolved in those La‐rich RE_6_(Fe, Al, Ga)_14_ phases generates the crystal defects of SFs. (3) Nd/Pr‐based GBDP induces the formation of NdPr‐rich RE_6_(Fe, Al, Ga)_14_ intergranular phase and NdPr‐rich magnetically hardening shell at an exceptionally long diffusion depth ≈2500 µm in the Al‐0.7 magnet, which finally results in the boosted magnetic performance of *H*_cj_ = 10.14 kOe and (*BH*)_max_ = 28.02 MGOe. These previously unattainable magnetic properties unequivocally demonstrate the pursuit of rationally designed MM–Fe–Al–Ga–B permanent material as a promising alternative with broad industrial application prospects.

## 5 Experimental Section

### Sample Preparation

Initial magnetic powders with the average compositions of MM_32_Fe_bal_Al*_x_* Ga_0.4_M_1.1_B_0.92_ (*x* = 0.2, 0.4, 0.7 and 1, M = Cu, Zr, wt%) were prepared by induction melting, strip casting, hydrogen decrepitating, and jet milling, using the Mischmetal (MM consists of 28.5 wt% La, 51.5 wt% Ce, 5.0 wt% Pr, 15.0 wt% Nd) from the Bayan Obo Mine. The average particle size of magnetic powders is ≈2.7 µm. The magnetic powders were then compressed and aligned under ≈5 MPa and ≈1.8 T, followed by isostatic compacting under ≈200 MPa. The green compacts were sintered for 3 h at ≈1050 °C in vacuum furnace with the pressure below 1 × 10^−2^ Pa to obtain the as‐sintered magnets, followed by two‐step annealing at ≈900 °C for 0.5 h and ≈500 °C for 2 h. For simplicity, those annealed magnets with respective Al contents of 0.2, 0.4, 0.7 and 1 wt% were designated as the Al‐0.2, Al‐0.4, Al‐0.7 and Al‐1 MM–Fe–Al–Ga–B magnets.

With the purpose of enhanced coercivity, Nd/Pr‐based alloys GBDP was carried out onto the Al‐0.7 as‐sintered magnet. The Nd/Pr‐based diffusion sources were prepared by hydrogen decrepitating and crushing into fine powders, then mixed with polyvinyl pyrrolidone and alcohol, and homogeneously placed over both pole surfaces of the Al‐0.7 as‐sintered magnet. The diffusion processing was carried out at ≈900 °C for 6–10 h, followed by annealing at ≈450 °C for 4 h to prepare the final Nd/Pr‐based GBDP MM–Fe–Al–Ga–B magnet.

To study the effects of dissolved Al concentration on the RE(Fe, Al)_2_ intergranular phase in MM–Fe–Al–Ga–B magnets, the ingots with a nominal composition of Nd_0.25_Ce_0.75_(Fe_1‐_ *_x_* Al*_x_*)_2_ (*x* = 0, 0.0625, 0.125 and 0.25, at%) were prepared via arc melting using high‐purity starting materials (≥99.99%). Then the ingots were wrapped into a Ta foil, sealed into an evacuated quartz tube, and annealed at ≈750 °C for 30 d under the protection of inert gas Ar, finally followed by ice‐water quenching.

### Sample Characterization

Room‐temperature demagnetization curves of the Al‐0.2, Al‐0.4, Al‐0.7 and Al‐1 magnets and the Nd/Pr‐based GBDP magnets were measured using a NIM‐62000 hysteresigraph analyzer. Thermomagnetic curves were measured upon heating to 300 °C with an external field of 1 kOe using vibrating sample magnetometer (VSM, LakeShore‐7410). Initial magnetization curves along easy and hard axes were measured using a physical property measurement system (PPMS, DYNACOOL). Phase constituents of the Nd_0.25_Ce_0.75_(Fe_1‐_ *_x_* Al*_x_*)_2_ (*x* = 0, 0.0625, 0.125 and 0.25, at%) homogeneously annealed alloys were conducted by an X‐ray diffractometer (XRD, Bruker‐D8) under a step of 0.02° staying for 4 s. Differential scanning calorimetry (DSC, NETZSCH STA‐449C) curves of these alloys were obtained upon heating to 1000 °C at a scan rate of 10 °C min^−1^ under Ar flow. Microstructural observations of the Al‐0.2, Al‐0.4, Al‐0.7 and Al‐1 magnets were conducted using a thermal field emission scanning electron microscope (SEM, ZEISS‐G300). Statistical analysis of elemental concentration of the Al‐0.4, Al‐0.7 and Al‐1 magnets were conducted via the supplemented energy dispersive X‐ray spectrometer (EDXS). Elemental distribution mappings were characterized using an electron probe microanalyzer (EPMA, Shimadzu‐1720) with wavelength dispersive X‐ray spectroscopy (WDXS). The area fractions of constituent phases were estimated using Image‐Pro Plus software. Transmission electron microscopy (TEM) lamellas were prepared from the Al‐0.7 and Al‐1 magnets via in‐situ focused ion beam lift‐out method using a FEI Helios G4 SEM/FIB DualBeam workstation. TEM and scanning transmission electron microscopy–energy dispersive X‐ray spectroscopy (STEM–EDXS) experiments were conducted on those TEM lamellas using a Thermo Scientific Talos F200X and a Thermo Spectra Ultra microscope with double spherical aberration correctors.

## Conflict of Interest

The authors declare no conflict of interest.

## Supporting information



Supporting Information

## Data Availability

The data that support the findings of this study are available from the corresponding author upon reasonable request.
