# Supplementary material for: A Promising MM–Fe–Al–Ga–B Permanent Material with Substantially Boosted Magnetic Performance
Source: Adv Sci (Weinh). 2025 Apr 17;12(23):2417679. doi: 10.1002/advs.202417679 (PMC12199320; doi:10.1002/advs.202417679)
Supplement: Supplementary file 1 — Supporting Information [file ADVS-12-2417679-s001.docx]

Supplementary information

**A Promising MM–Fe–Al–Ga–B Permanent Material with Substantially Boosted Magnetic Performance**

Wang Chen^a,b,+^, Shaoqing Ren^a,b,+^, Jiaying Jin^a,b,^*, Liang Zhou^b^, Bo Xin^a^, Mingjing Zhao^a^, Mengfan Bu^b^, Xu Li^b^, Chen Wu^b^, Mi Yan^a,b,^*

^a^*State Key Laboratory of Baiyunobo Rare Earth Resource Researches and Comprehensive Utilization, Baotou Research Institution of Rare Earths, Baotou 014030, China*

^b^*State Key Laboratory of Silicon and Advanced Semiconductor Materials, School of Materials Science and Engineering, Key Laboratory of Novel Materials for Information Technology of Zhejiang Province, Zhejiang University, Hangzhou 310027, China*

^+^ These authors contributed equally to this work.

* Corresponding authors. jinjy@zju.edu.cn (Dr. J. Jin), mse_yanmi@zju.edu.cn (Prof. M. Yan)


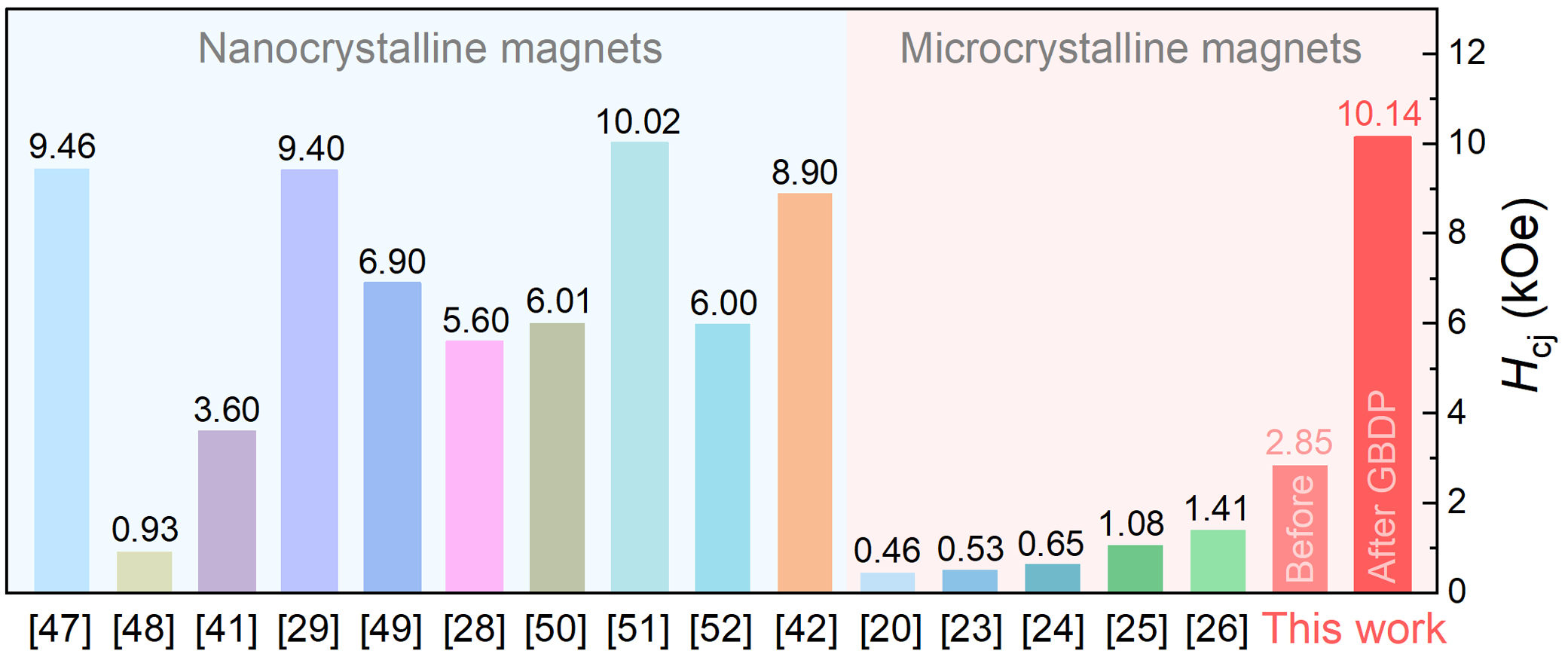


**Figure S1.** Benchmark *H*_cj_ of the present Al-0.7 magnet before and after GBDP (marked by red columns) compared with a wide range of other nanocrystalline^[28,29,41,42,47-52]^ and microcrystalline^[20,23-26]^ MM–Fe–B magnets.


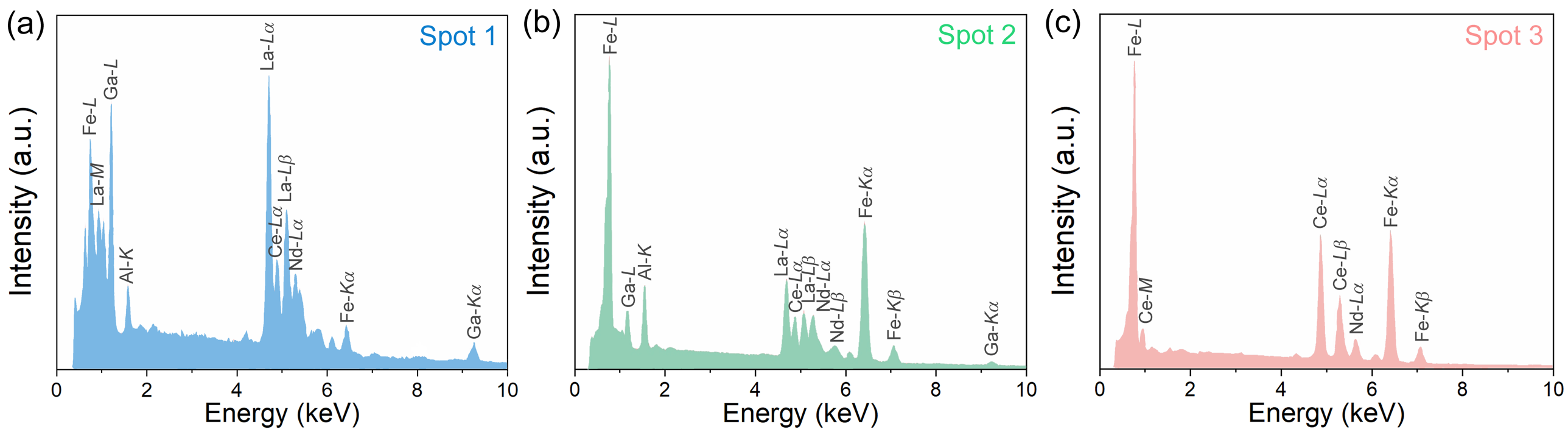


**Figure S2.** Corresponding EDXS spectra at the typical intergranular regions in **Figure** **5**: (a) Spot 1 relating to the LaCe-rich RE–(Al, Ga) intergranular phase, (b) Spot 2 relating to the La-rich RE_6_(Fe, Al, Ga)_14_ intergranular phase, (c) Spot 3 relating to the Ce-rich REFe_2_ intergranular phase.


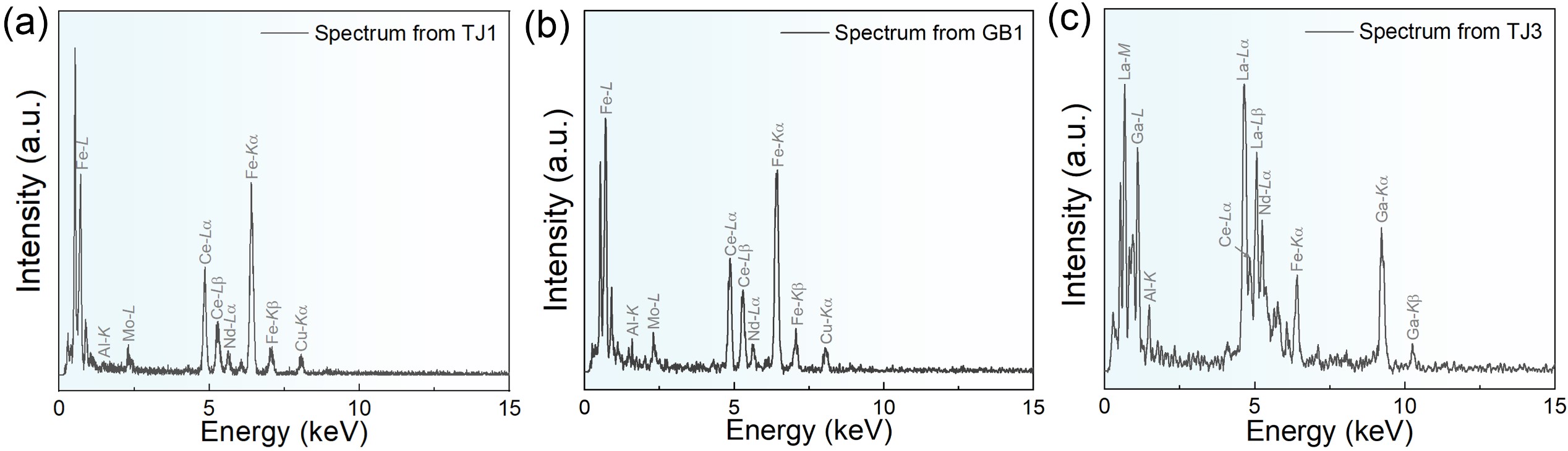


**Figure S3.** Corresponding EDXS spectra at the typical intergranular regions in the Al-0.7 magnet: (a) TJ1 in **Figure** 6h, (b) GB1 in **Figure** 6i, (c) TJ3 in **Figure** 6l.


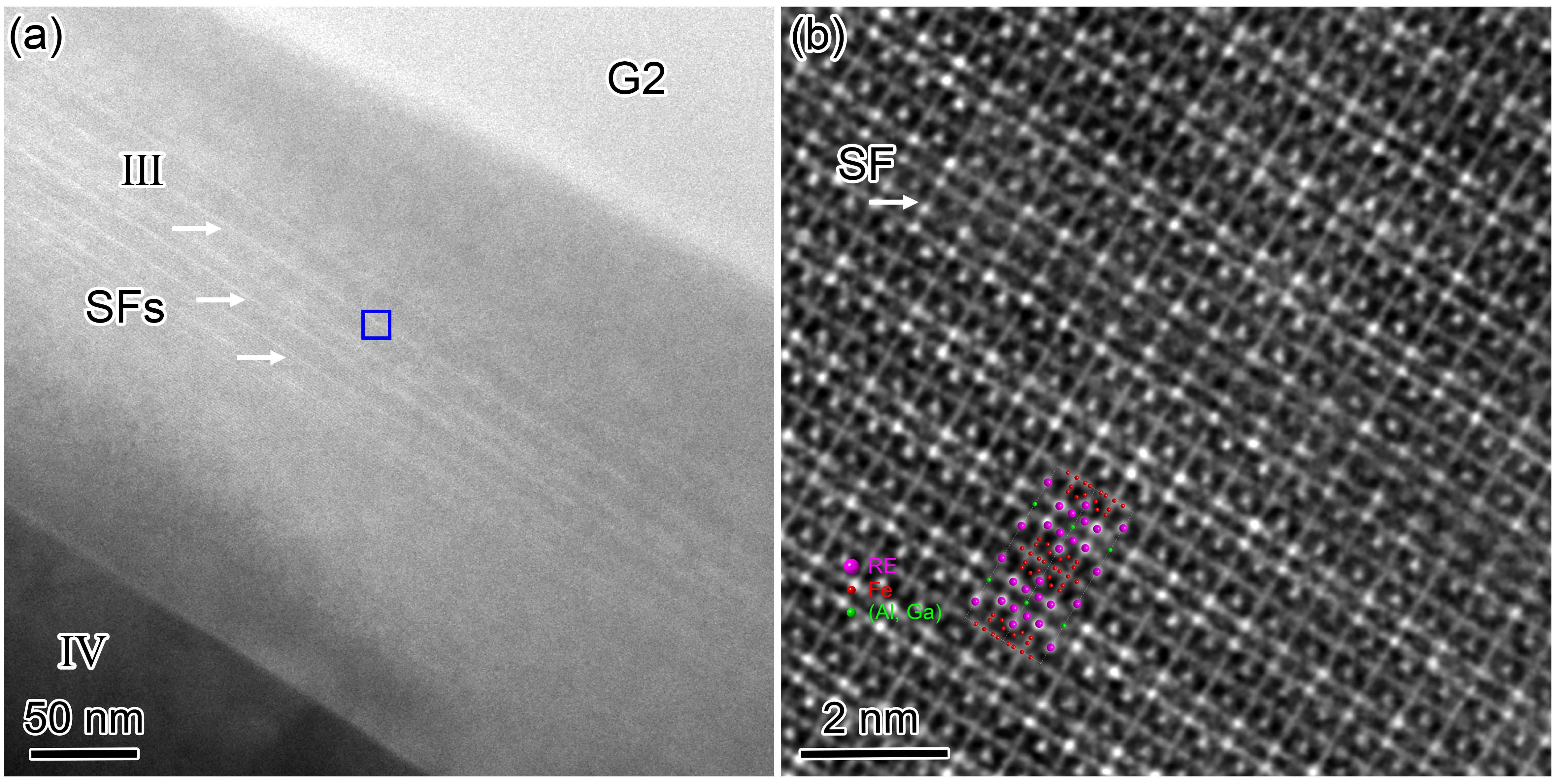


**Figure S4.** STEM analysis of another typical region in the Al-1 magnet. (a) HAADF-STEM image of the Ⅳ/Ⅲ/G2 interface. (b) Atomic-scale HAADF-STEM image of the blue square region in (a) with obvious SF.


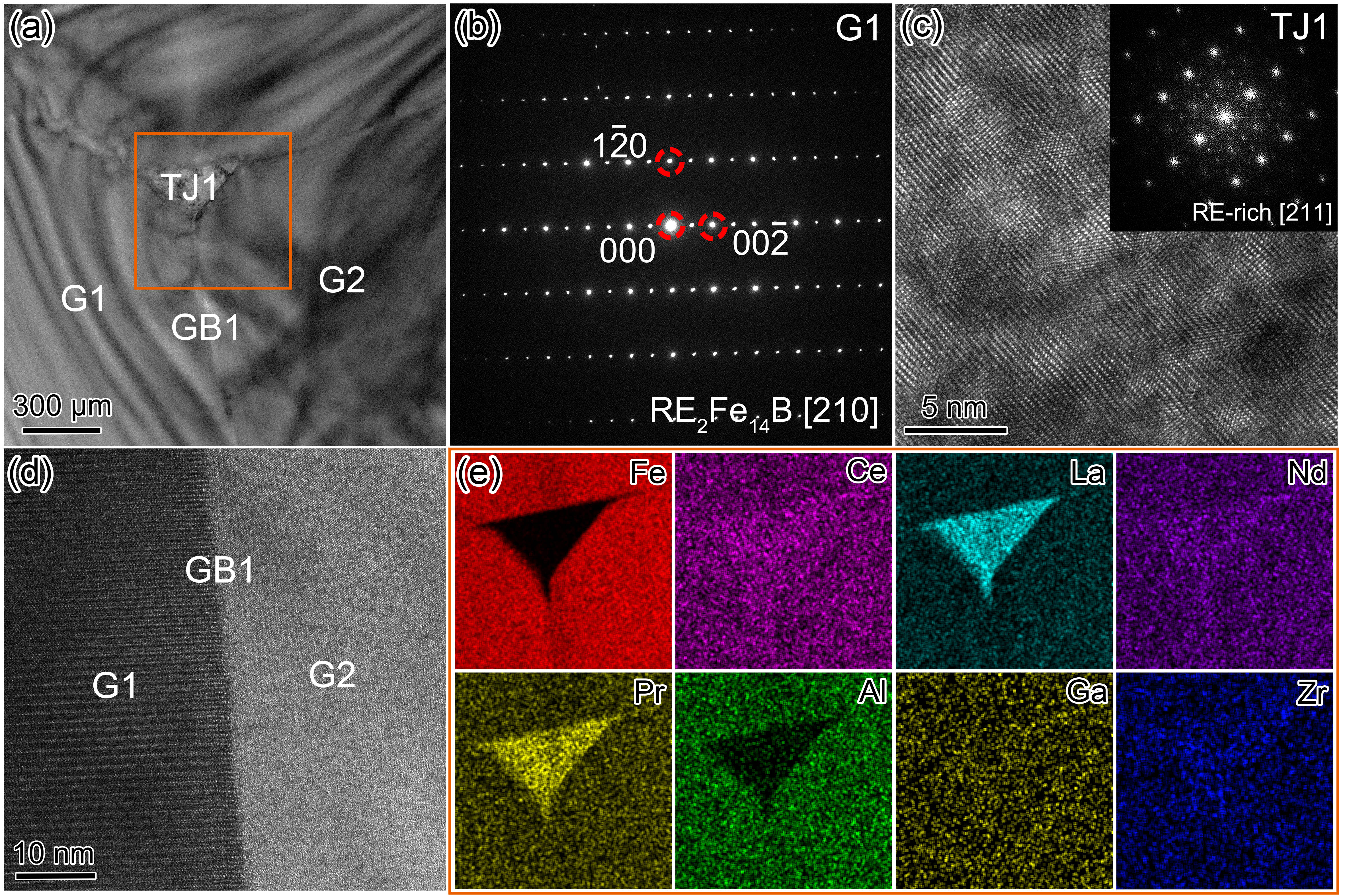


**Figure S5.** TEM analysis of another typical region in the Al-1 magnet. (a) Typical BFI. (b) SAED pattern of G1. (c) HRTEM image of TJ1, the upper-right inset shows the corresponding FFT pattern. (d) HRTEM image of G1/GB1/G2 interface, showing the directly contacted matrix phase grains without thick GB layers. (e) Elemental distribution mappings of Fe, Ce, La, Nd, Pr, Al, Ga and Zr taken from the square region in (a).


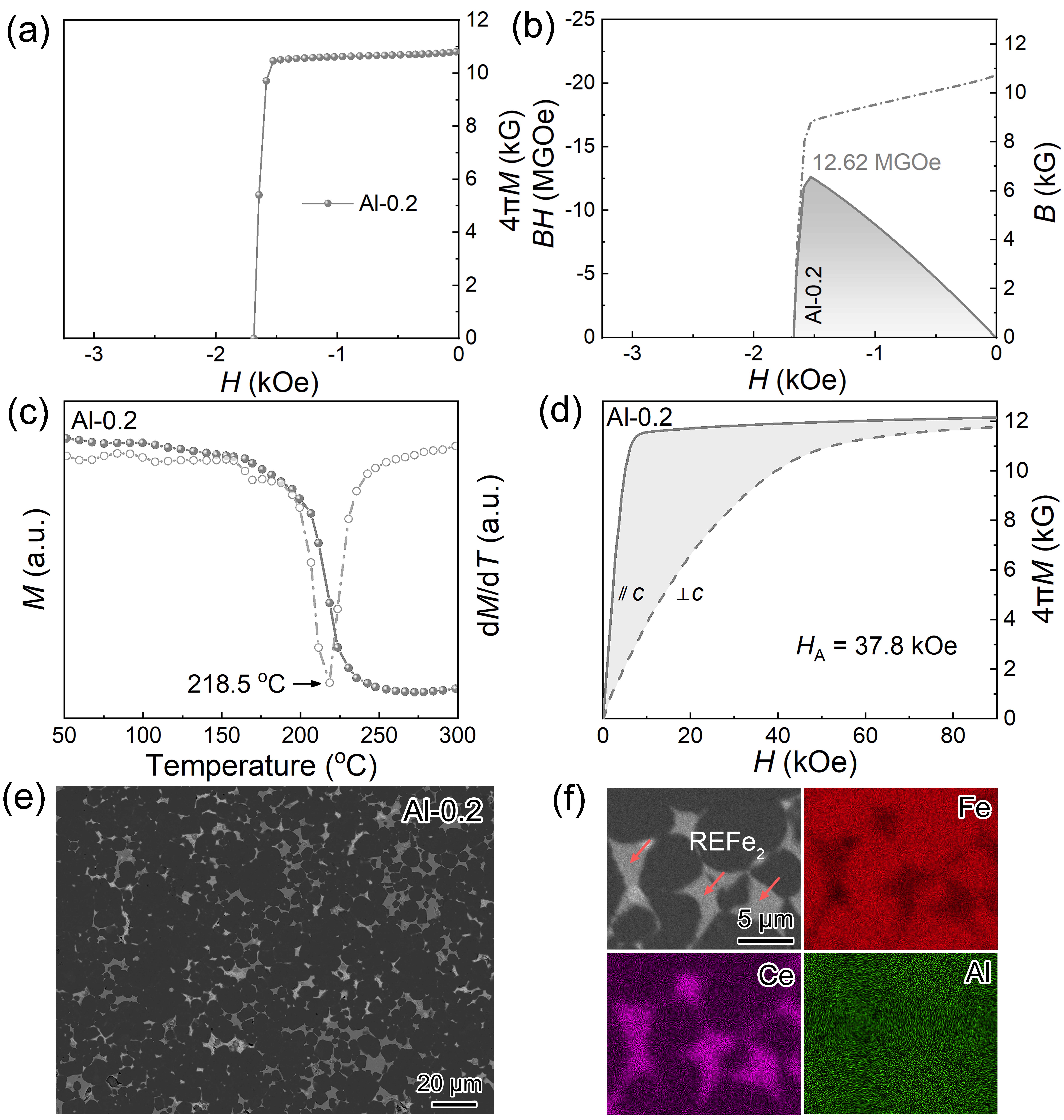


**Figure S6.** Magnetic performance and microstructural characterization of the Al-0.2 MM–Fe–Al–Ga–B magnet: (a) *J*-*H* and (b) *B*-*H* demagnetization curves. (c) Normalized *M*-*T* and derived d*M*/d*T*-*T* curves. (d) Initial *M*-*H* curves along easy (∥*c* axis) and hard (⊥*c* axis) axes to obtain the intrinsic *H*_A_. (e) Low-magnification and (f) high-magnification BSE SEM images, with corresponding EDXS mappings.


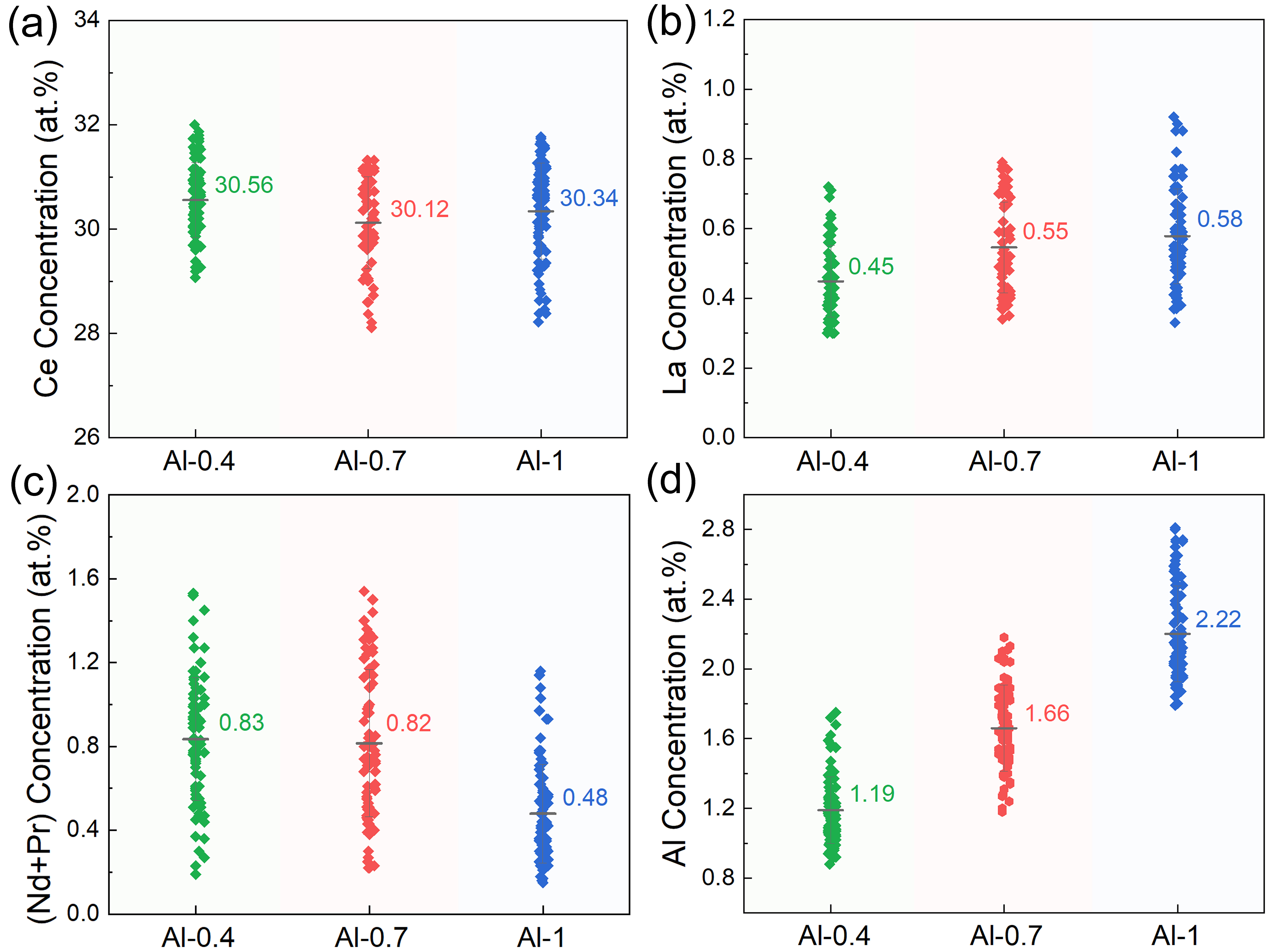


**Figure S7.** Statistical analysis of (a) Ce, (b) La, (c) (Nd+Pr) and (d) Al concentration gradients by detecting approximately 100 regions of Ce-rich REFe_2_ intergranular phase for each Al-0.4, Al-0.7 and Al-1 magnet.


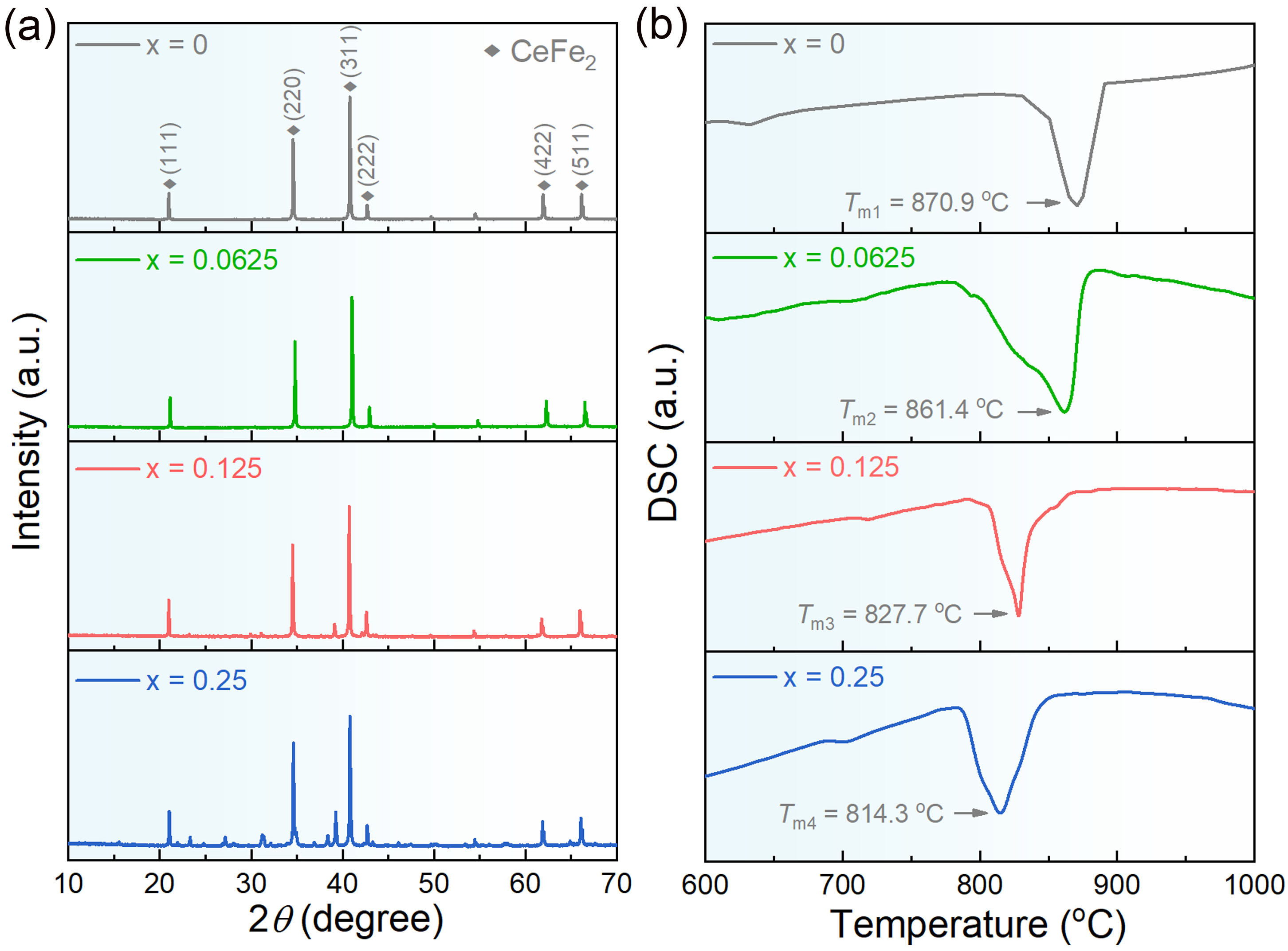


**Figure S8.** (a) Step-scanned XRD patterns and (b) DSC curves of Nd_0.25_Ce_0.75_(Fe_1-_*_x_*Al*_x_*)_2_ (*x* = 0, 0.0625, 0.125 and 0.25, at.%) homogeneous annealed alloys, indicating the lower melting point of Ce-rich RE(Fe, Al)_2_ phase with increasing Al content.


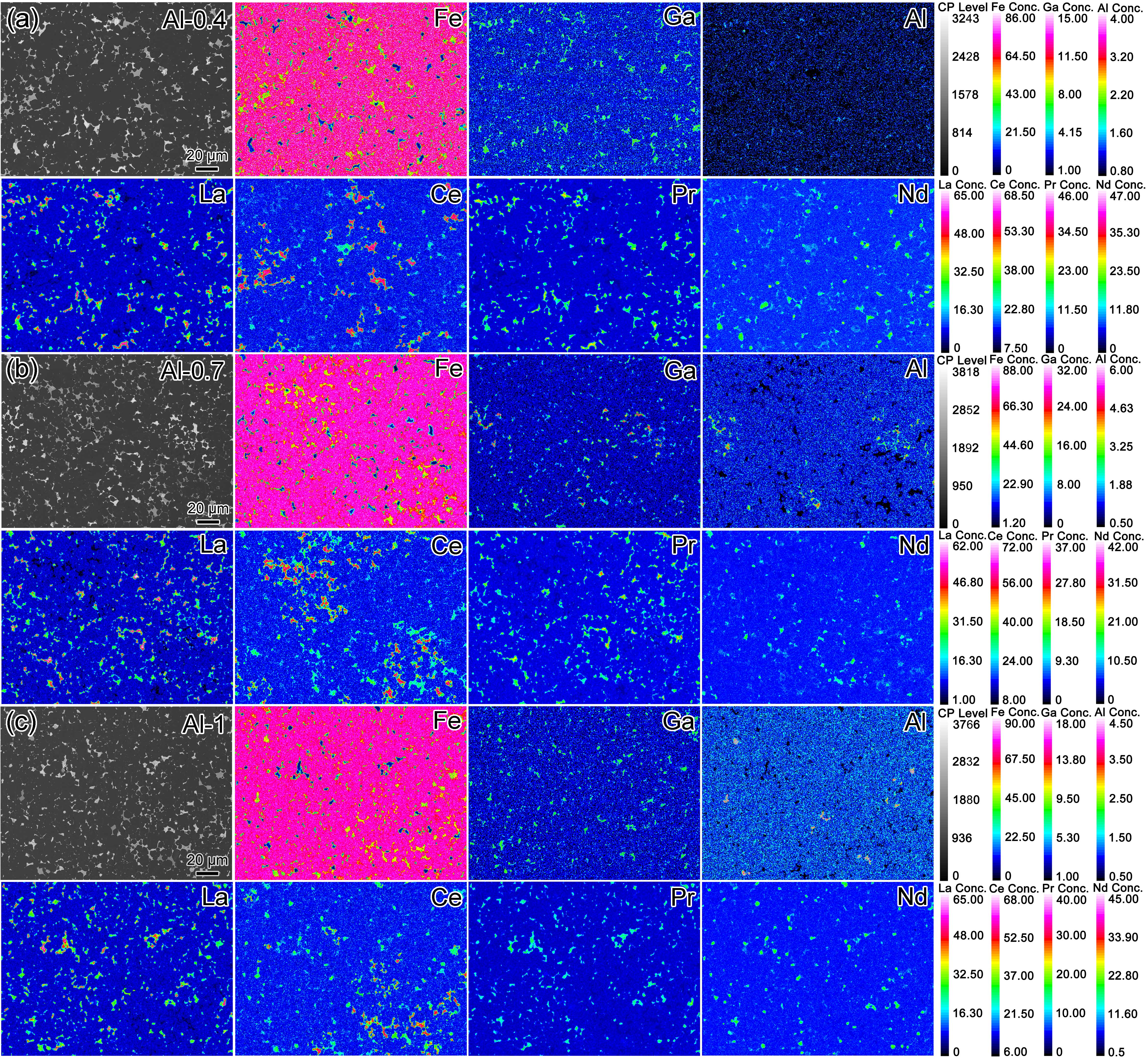


**Figure S9.** Corresponding low-magnification EPMA images for (a) Al-0.4, (b) Al-0.7 and (c) Al-1 magnets shown in Figure 4.


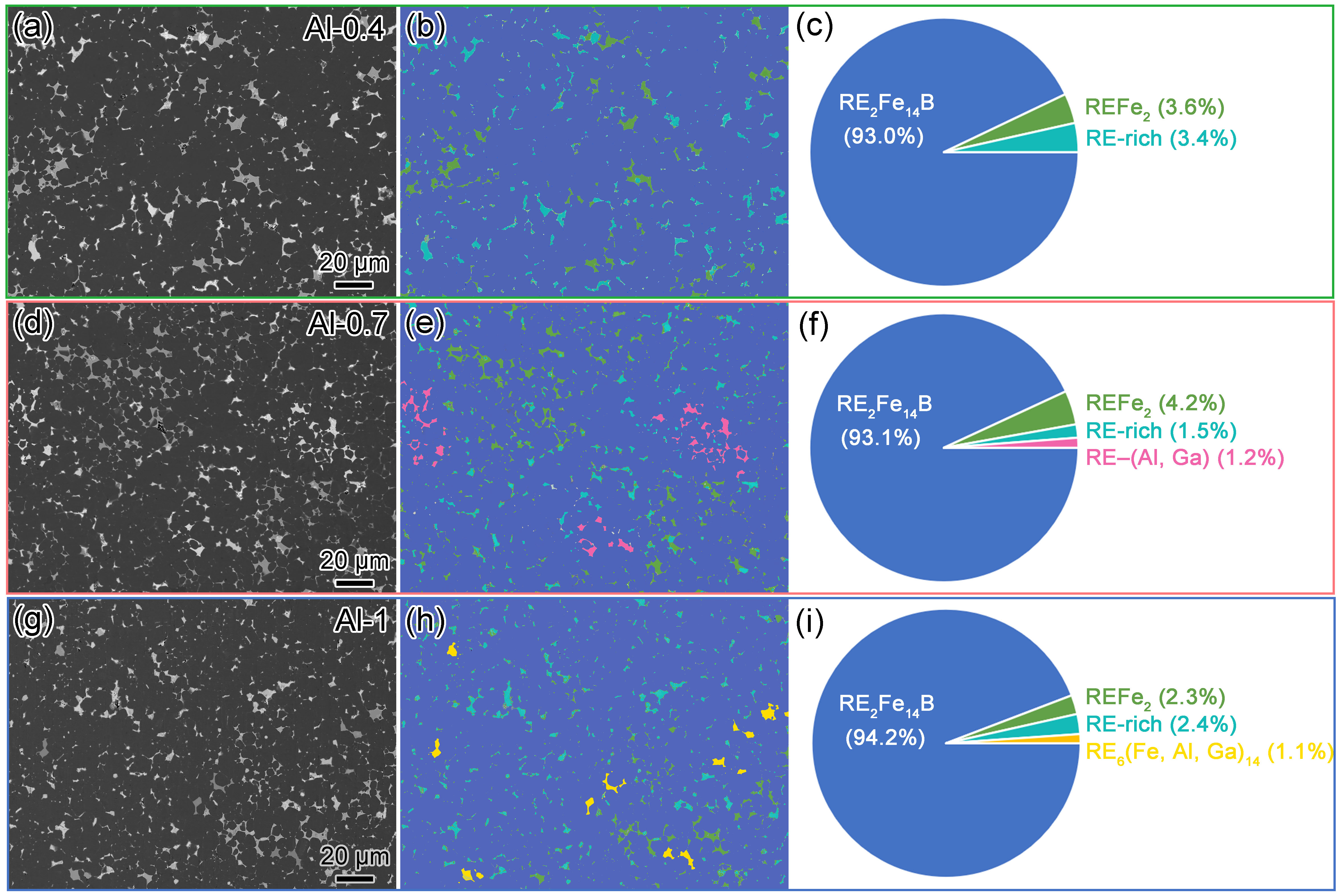


**Figure S10.** Low-magnification BSE SEM images, and corresponding areal fractions of each constituent phase depicted by pie graphs using Image-Pro Plus software for (a-c) Al-0.4, (b-f) Al-0.7 and (g-i) Al-1 magnets. Note that each phase was identified by the EPMA analysis presented in Figure S9.


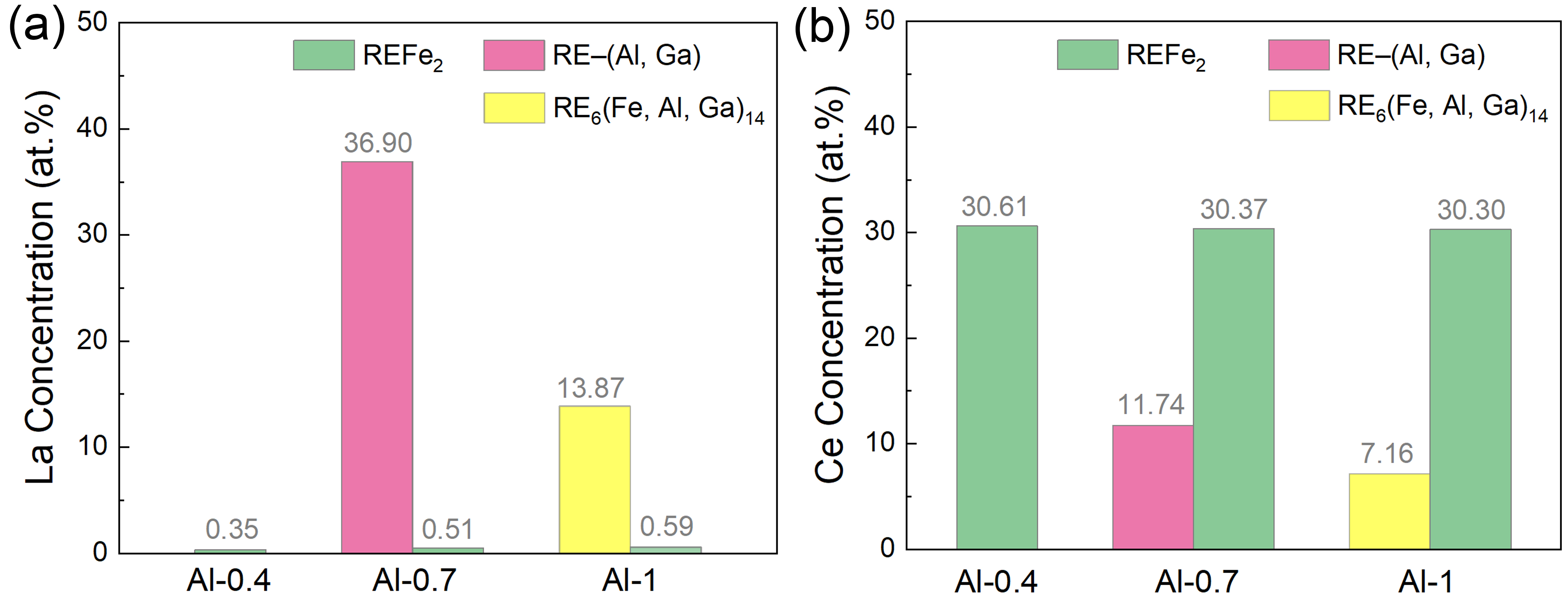


**Figure S11.** EDXS analysis of (a) La and (b) Ce concentrations within the three types of intergranular phases for each Al-0.4, Al-0.7 and Al-1 magnet, including the REFe_2_ phase, RE–(Al, Ga) phase and RE_6_(Fe, Al, Ga)_14_ phase.


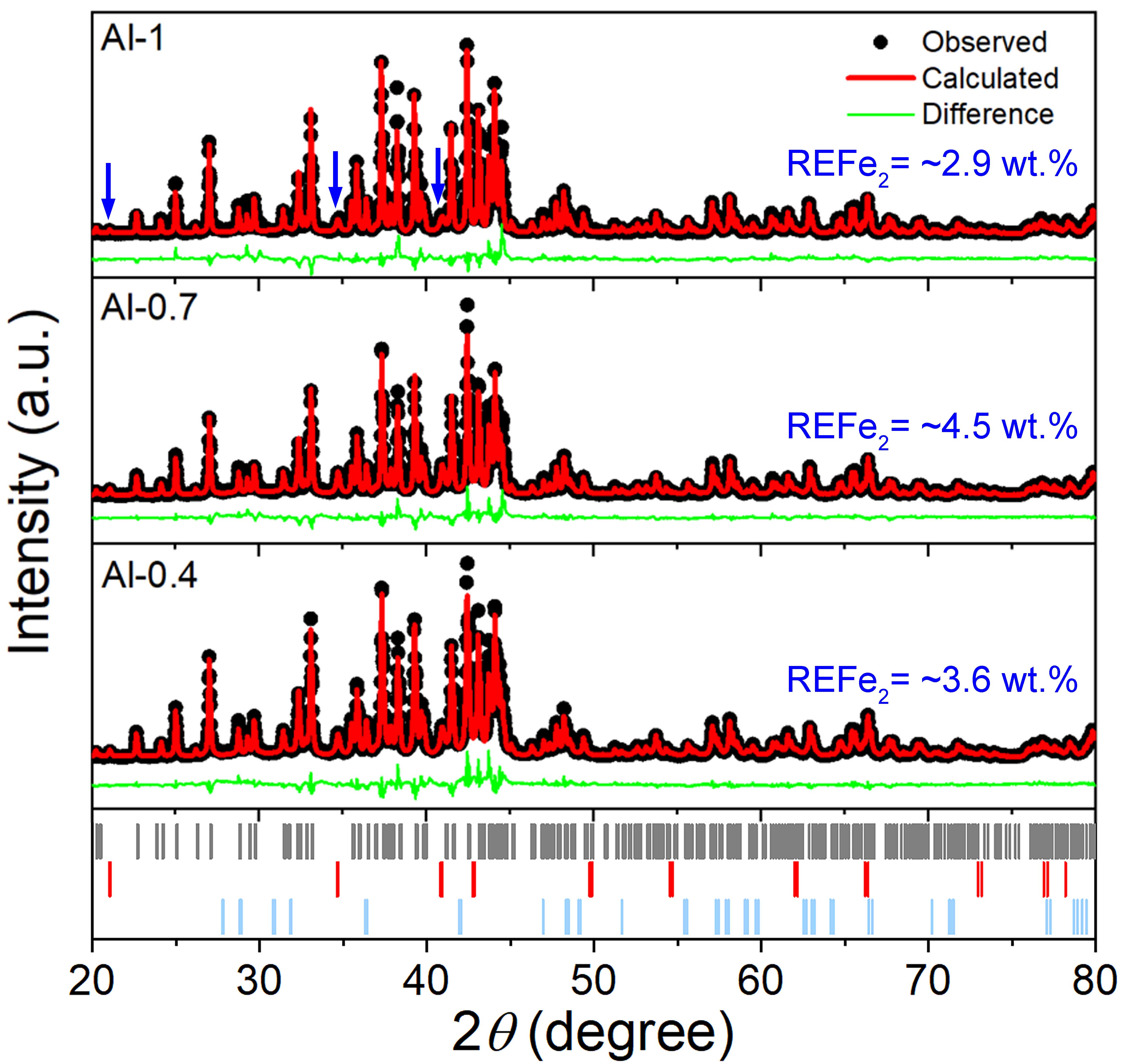


**Figure S12.** Rietveld refinement of step-scanned XRD patterns for Al-0.4, Al-0.7 and Al-1 magnets in the form of magnetic powders. Bottom ticks mark the characteristic Bragg positions of RE_2_Fe_14_B, REFe_2_ and RE-rich phases in gray, red and blue colors, respectively.

**Table S1.** Summary of the nominal composition and magnetic performance of MM–Fe–B magnets in previous publications.^[20,23-26,28,29,41,42,47-52]^

| Refs | MM | | | | | | RE (wt.%) | *H*_cj_ (kOe) | (*BH*)_max_ (MGOe) |
| --- | --- | --- | --- | --- | --- | --- | --- | --- | --- |
|  | La (wt.%) | Ce (wt.%) | Pr (wt.%) | Nd (wt.%) | Other (wt.%) | (La+Ce)/RE (%) |  |  |  |
| [47] | 28.63 | 50.13 | 4.81 | 16.38 | / | 78.80 | 30.28 | 9.46 | 0.89 |
| [48] | 23.17 | 53.96 | 5.16 | 17.60 | / | 77.21 | 29.65 | 0.93 | 4.00 |
| [41] | 26.09 | 51.90 | 5.33 | 14.25 | 2.43 | 79.93 | 28.21 | 3.60 | 6.80 |
| [29] | 31.31 | 47.10 | 4.46 | 14.78 | 2.35 | 80.60 | 34.34 | 9.40 | 8.10 |
| [49] | 25 | 50 | 6 | 19 | / | 75.00 | 28.42 | 6.90 | 8.50 |
| [28] | 26.09 | 51.90 | 5.33 | 14.25 | 2.43 | 79.93 | 33.89 | 5.60 | 8.90 |
| [50] | 27.01 | 51.52 | 4.09 | 16.21 | 1.17 | 79.46 | 32.17 | 6.01 | 9.22 |
| [51] | 23.8 | 47.7 | 7.1 | 21.3 | / | 71.57 | 28.1 | 10.02 | 9.75 |
| [52] | 28.27 | 50.46 | 5.22 | 15.66 | 0.39 | 79.04 | 28.42 | 6.00 | 12.20 |
| [42] | 25 | 53 | 5 | 17 | / | 78 | 33.76 | 8.9 | 13.84 |
| [20] | 27.06 | 51.46 | 5.22 | 16.16 | 0.1 | 78.60 | 32.48 | 0.46 | 2.40 |
| [23] | 23-35 | 45-60 | 3-7 | 9-20 | / | 81.5 | 30.3 | 0.53 | 2.71 |
| [24] | 28.0 | 52.0 | 5.1 | 14.7 | 0.2 | 80.16 | 30.27 | 0.65 | 2.72 |
| [25] | 28.0 | 52.0 | 5.1 | 14.7 | 0.2 | 80.16 | 30.23 | 1.08 | 7.60 |
| [26] | 25 | 55 | 5 | 15 | / | 80 | 30.8 | 1.41 | 8.05 |

**Table S2.** Inductively coupled plasma–atomic emission spectroscopy (ICP–AES) analysis of the Al-0.4, Al-0.7 and Al-1 magnets.

| Magnet | La (wt.%) | Ce (wt.%) | Pr (wt.%) | Nd (wt.%) | Fe (wt.%) | Al (wt.%) | Ga (wt.%) |
| --- | --- | --- | --- | --- | --- | --- | --- |
| Al-0.4 | 7.58 | 15.50 | 1.83 | 5.83 | 65.29 | 0.39 | 0.41 |
| Al-0.7 | 8.44 | 16.11 | 1.58 | 5.02 | 66.54 | 0.73 | 0.48 |
| Al-1 | 8.26 | 15.76 | 1.62 | 5.18 | 64.57 | 0.98 | 0.47 |

**Table S3.** Summary of the chemical composition of the typical intergranular phases in the Al-0.4, Al-0.7, Al-1 magnets, as well as the Nd/Pr-based GBDP magnet.

| Magnet | Phase | La (at.%) | Ce (at.%) | Pr (at.%) | Nd (at.%) | Fe (at.%) | Al (at.%) | Ga (at.%) | Others (at.%) |
| --- | --- | --- | --- | --- | --- | --- | --- | --- | --- |
| Al-0.4 | Ce-rich REFe_2_ | 0.35 | 30.61 | 0.29 | 0.87 | 64.59 | 1.17 | 0.49 | 1.63 |
| Al-0.7 | Ce-rich REFe_2_ | 0.51 | 30.37 | 0.28 | 0.88 | 64.48 | 1.60 | 0.34 | 1.54 |
|  | LaCe-rich RE–(Al, Ga) | 36.90 | 11.74 | 2.64 | 6.37 | 9.70 | 7.73 | 23.05 | 1.87 |
| Al-1 | Ce-rich REFe_2_ | 0.59 | 30.30 | 0.10 | 0.56 | 63.29 | 2.29 | 0.53 | 2.34 |
|  | La-rich RE_6_(Fe, Al, Ga)_14_ | 13.87 | 7.16 | 0.50 | 4.56 | 55.08 | 12.54 | 4.96 | 1.33 |
| Nd/Pr-based GBDP | NdPr-rich RE_6_(Fe, Al, Ga)_14_ | 1.52 | 3.43 | 15.56 | 6.54 | 62.92 | 4.44 | 5.14 | 0.45 |
|  | NdPr-rich RE–(Al, Ga) | 3.93 | 4.81 | 34.40 | 14.03 | 12.62 | 3.42 | 24.25 | 2.54 |
